# Structural basis of interaction between dimeric cyclophilin 1 and Myb1 transcription factor in *Trichomonas vaginalis*

**DOI:** 10.1038/s41598-018-23821-5

**Published:** 2018-04-03

**Authors:** Tesmine Martin, Yuan-Chao Lou, Chun-Chi Chou, Shu-Yi Wei, Sushant Sadotra, Chao-Cheng Cho, Meng-Hsuan Lin, Jung-Hsiang Tai, Chun-Hua Hsu, Chinpan Chen

**Affiliations:** 1 Institute of Biomedical Sciences, Academia Sinica, Taipei, 115 Taiwan; 2 Chemical Biology and Molecular Biophysics, Taiwan International Graduate Program, Academia Sinica., Taipei, 115 Taiwan; 3 Department of Chemistry, National Tsing Hua University, Hsinchu, 300 Taiwan; 4 Institute of Bioinformatics and Structural Biology, National Tsing Hua University, Hsinchu, 300 Taiwan; 5 Genome and Systems Biology Degree Program, National Taiwan University and Academia Sinica, Taipei, 106 Taiwan; 6 Department of Agricultural Chemistry, National Taiwan University, Taipei, 106 Taiwan

## Abstract

Cyclophilin 1 (*Tv*CyP1), a cyclophilin type peptidyl-prolyl isomerase present in the human parasite *Trichomonas vaginalis*, interacts with Myb1 and assists in its nuclear translocation. Myb1 regulates the expression of *ap65-*1 gene that encodes for a disease causing cytoadherence enzyme. Here, we determined the crystal structures of *Tv*CyP1 and its complex with the minimum *Tv*CyP1-binding sequence of Myb1 (Myb1^104–111^), where *Tv*CyP1 formed a homodimer, unlike other single domain cyclophilins. In the complex structure, one Myb1^104–111^ peptide was bound to each *Tv*CyP1 protomer, with G106-P107 and Y105 fitting well into the active site and auxiliary S2 pocket, respectively. NMR data further showed that *Tv*CyP1 can catalyze the *cis/trans* isomerization of P107 in Myb1^104–111^. Interestingly, in the well-folded Myb1 protein (Myb1^35–141^), the minimum binding sequence adopted a different conformation from that of unstructured Myb1^104–111^ peptide, that could make P107 binding to the active site of *Tv*CyP1 difficult. However, NMR studies showed that similar to Myb1^104–111^ peptide, Myb1^35–141^ also interacted with the active site of *Tv*CyP1 and the dynamics of the Myb1^35–141^ residues near P107 was reduced upon interaction. Together, the structure of *Tv*CyP1 and detailed structural insights on *Tv*CyP1-Myb1 interaction provided here could pave the way for newer drugs to treat drug-resistant strains.

## Introduction

Cyclophilins are a family of ubiquitous and versatile enzymes exhibiting peptidyl-prolyl isomerase (PPIase) activity^1^. They perform a wide variety of biological functions either by the catalysis of *cis/trans* isomerization of prolyl peptide bonds or as binding partners of biological substrates. The various biological functions of cyclophilins include, but not limited to protein folding and trafficking, immune response, signal transduction, viral infection and transcription regulation^2,3^. Human cyclophilin A (*H*cypA) is the well-studied cyclophilin that is found to interact with several proteins leading to various diseases including HIV infection and cancer^3^. In spite of identifying a large number of interacting proteins of *H*cypA, the underlying mechanism of *H*cypA action and the physiological outcome of interactions are not known in most cases.

Catalytically active cyclophilins with a single cyclophilin domain reported till now are monomeric in nature. The PPIase activity of cyclophilins can be inhibited by cyclosporin A (CsA), sanglifehrin A (SfA) or their non-immunosuppressive analogues by binding to the active site of cyclophilins^4,5^. Besides the active site where substrate proline binds and catalysis occurs, cyclophilins also possess an auxiliary S2 pocket guarded by “gatekeeper residues”, where the preceding residues of the isomerized proline interact^6^. The “gatekeeper region” of the S2 pocket shows maximum chemical diversity among various isoforms of cyclophilins. Hence, the region surrounding the S2 pocket is the most ideal site for designing drugs with enhanced specificity and binding affinity for particular isoforms of the cyclophilin family.

Parasitic cyclophilins influence different stages of a parasite’s development, and CsA or its analogues have been found with anti-parasitic activity^7,8^. Most of the parasitic cyclophilins reported to date possess high sequence similarity and identity to *H*cypA^9,10^. In most cases, the biological substrates of these cyclophilins are unknown and extensive structural studies on cyclophilin- substrate interactions have not been reported.

*Tv*CyP1 is a cyclophilin present in the parasite *Trichomonas vaginalis* (*T. vaginalis*) showing high sequence identity to *H*cypA. *T. vaginalis* is the most common, sexually transmitted, non-viral pathogen infecting humans, causing Trichomoniasis. The infection causes several adverse reproductive outcomes and even cervical and prostate cancers^11–14^. *T. vaginalis* also maintains a symbiotic relationship with various other parasitic organisms, which can eventually lead to an inflammatory response or resistance to metronidazole (MTZ)^15^. The availability of only a single class of drugs to treat the disease is a major concern for public health, with the emergence of drug resistant strains, especially due to the higher transmission of HIV and HPV in infected individuals^16–19^.

Cytoadherence is one of the most important contact-dependent mechanisms by which *T. vaginalis* begins and continues chronic infection. Among the several hydrogenosomal enzymes that also function as adhesins, AP65, encoded by *ap65-1* gene, plays an important role in cytoadherence of the parasite to human vaginal epithelial cells^20,21^. AP65 is a 65 kDa hydrogenosomal malic enzyme primarily involved in carbohydrate metabolism, but also act as adhesin at the surface of the parasite. The temporal and iron-inducible expression of AP65 is regulated by the coordinated actions of three Myb-like transcription factors, Myb1, Myb2 and Myb3, by their differential and competitive selection of the entry sites in the promoter of *ap65-1* gene^22–24^. Nuclear import of these Myb proteins is a crucial checkpoint that regulates transcription, which in Myb2 and Myb3 occurs via their own DNA binding domains (DBDs)^25,26^. However, for Myb1, the nuclear translocation is regulated by *Tv*CyP1. The interaction of *Tv*CyP1 with Myb1 leads to the cytoplasmic release of Myb1 from membrane-bound vesicles, an important step preceding translocation to nucleus. We also identified a minimum *Tv*CyP1 binding sequence in Myb1 (Myb1^104–111^), in which ^105^YGP^107^ was found critical in *Tv*CyP1 interaction. Mutations of G106 and P107 in Myb1 changed the cytoplasmic retention and nuclear translocation of overexpressed Myb1 protein^27^.

In this study, we determined the crystal structures of *Tv*CyP1 in the absence and presence of Myb1^104–111^. Unlike other dimeric or multimeric single domain cyclophilins^28,29^, *Tv*CyP1 formed a homodimer without hindering the substrate binding site and existed as a dimer even in solution. In the complex structure, P107 of one Myb1^104–111^ molecule was bound to the active site of each *Tv*CyP1 protomer. Presence of tyrosine preceding the Gly-Pro bond in Myb1^104–111^ allowed optimal interaction with the S2 pocket. Using NMR, we have also confirmed the catalytic behavior of *Tv*CyP1 and inhibition of catalysis by CsA. Although our attempts to crystallize the *Tv*CyP1–Myb1^35–141^ complex were not successful, with NMR and site-directed mutagenesis, we showed that P107 of Myb1^35–141^ also interacts with the active site of *Tv*CyP1 similar to Myb1^104–111^ peptide fragment. NMR Carr–Purcell–Meiboom–Gill (CPMG) relaxation dispersion experiments suggested that many Myb1^35–141^ residues exhibit millisecond time-scale dynamics and these conformational exchanges were notably reduced for the residues surrounding P107 upon addition of *Tv*CyP1. By and large, our study reports for the first time the structure of a catalytically active dimeric cyclophilin possessing only a single cyclophilin domain and provides structural details of its interaction with an intriguing biological substrate. The data shown here could provide further insights into the design of newer drugs to treat Trichomoniasis.

## Results

### *Tv*CyP1 is a divergent, single domain cyclophilin and forms a stable dimer in solution

*Tv*CyP1 is a single domain cyclophilin that shows high sequence identity to human and parasitic cyclophilins, all of which are monomers (Supplementary Fig. S1). Like *Caenorhabditis elegans* Cyp3 (*C*cyp3; PDB: 1DYW) and *Brugia malayi* CypB (BcypB; PDB: 4JCP), *Tv*CyP1 is a divergent loop cyclophilin and possesses an additional loop in the residues 49–55 (KSGMPLS)^30^. Although *Tv*CyP1 contains the conserved E84, which helps to lock and hitch the loop into a certain conformation, the divergent loop shows variation from the consensus loop sequence (**GK*LH), observed in all other divergent cyclophilins. Because of the presence of serine instead of a highly conserved H54 seen in *C*cyp3, *Tv*CyP1 lacks the metal ion coordination site formed by the imidazole sidechain of H54 and sulfhydryl groups of C168 and C40 (numbering based on 1DYW)^30^.

**Figure 1:**
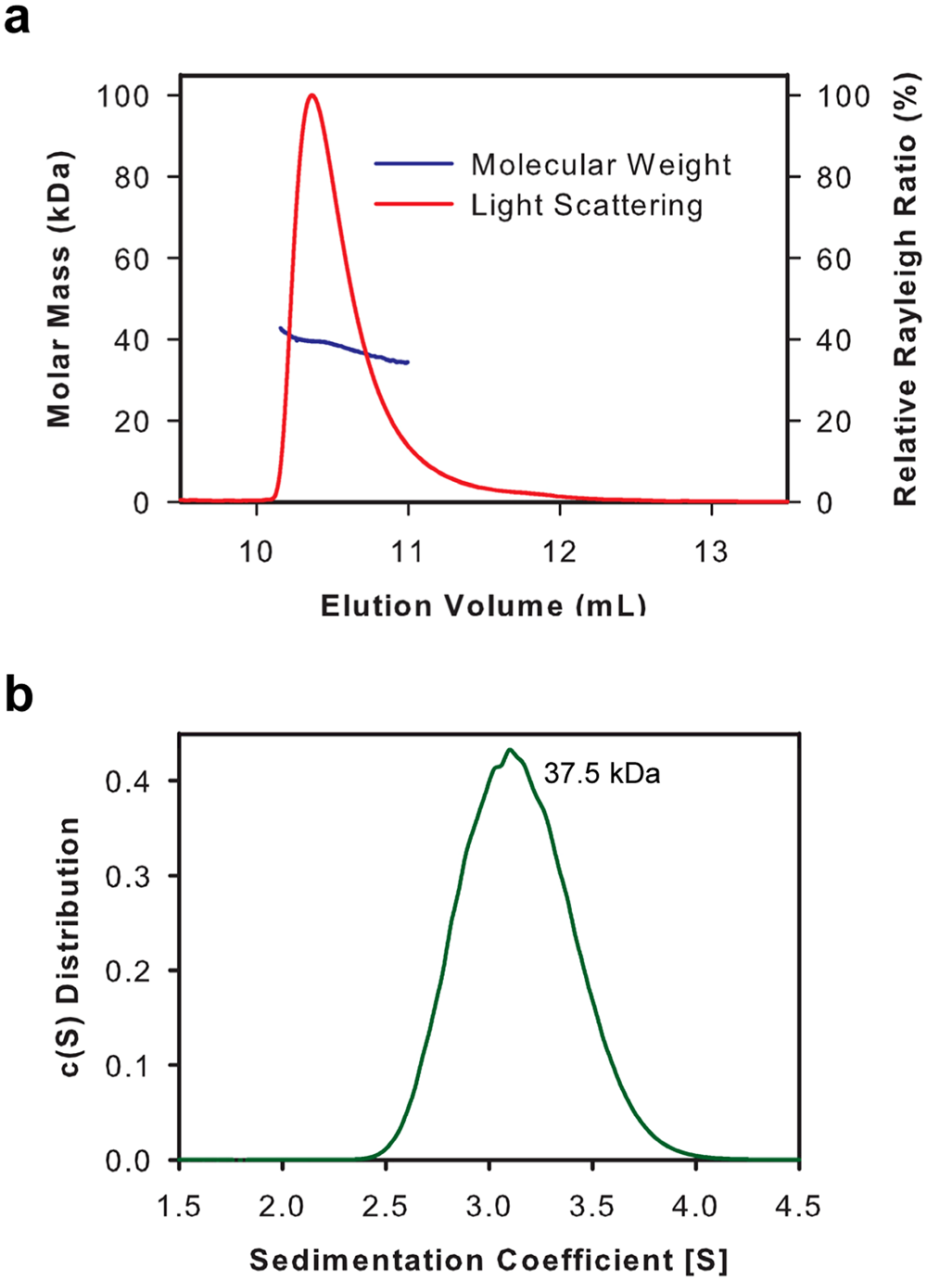
Dimeric nature of *Tv*CyP1 in solution. (**a**) SEC-MALS analysis of 3 mg/mL *Tv*CyP1 showing the presence of a dimer in solution. Molecular weight of *Tv*CyP1 monomer is 19.25 kDa. (**b**) AUC-SV profile of *Tv*CyP1 (30 μM concentration) showing continuous sedimentation coefficient distribution. The experimental data analysis by using Sedfit yielded an estimated molecular weight (MW) of 37.5 kDa, very close to the dimeric MW (38.5 kDa).

To evaluate whether *Tv*CyP1 exists as a monomer or dimer in solution, we performed size-exclusion chromatography with multi-angle light scattering (SEC-MALS) and analytical ultracentrifugation–sedimentation velocity (AUC-SV). The results from both the experiments confirmed that the protein exists as a dimer in solution (Fig. 1a,b). This is in contrast to the behavior of all other single domain cyclophilins in solution. In addition, the sensitivity of 3D triple resonance experiments was not sufficient to assign any of the side chain carbons and protons, possibly because of shorter transverse relaxation rate (T_2_), typical of a larger-sized protein. Presence of only a single set of peaks in [^1^H, ^15^N]-transverse relaxation optimized spectroscopy–heteronuclear single quantum coherence (TROSY-HSQC) spectrum further confirmed the symmetric structure of *Tv*CyP1 in solution (Supplementary Fig. S2).

### Crystal structure of *Tv*CyP1

To elucidate the structure of *Tv*CyP1, we crystallized it and obtained hexagonal crystals that gave optimal quality X-ray diffraction and belonged to the space group *P*6_1_22 with the following unit cell dimensions: a = b = 38.51 Å, c = 365.70 Å, and α =  β = 90°, γ = 120°. The structure of *Tv*CyP1 was solved by molecular replacement by using the structure of *C*cyp3 (PDB: 1DYW)^30^ as a search model, which shares 70% and 82% sequence identity and similarity, respectively, with *Tv*CyP1. The final crystal structure was refined to 2.27 Å resolution with R_work_ and R_free_ values of 21.20% and 27.80%, respectively (Table 1).

**Table 1:** Data collection and refinement statistics of *Tv*CyP1 apo and peptide-bound structures.

**Crystal**	**Apo form**	**Peptide-bound form**
Crystal parameters		
Space group	*P6*_*1*_22	*P2*_1_2_1_2_1_
Unit cell parameters		
a, b, c (Å)	38.51; 38.51; 365.70	37.79; 78.07; 117.71
α, β, γ (°)	90, 90, 120	90, 90, 90
Monomers per asymmetricunit cell	1	2
Data collection		
Wavelength (Å)	0.9998	0.9762
Resolution range (Å)	29.25–2.28 (2.35–2.28)	29.45–2.06 (2.14–2.06)
Unique no. of reflections	8106	21875
Total no. of reflections	29747	115767
I/σ^a^	6.18 (2.70)	9.81 (2.46)
*R*_merge_^a,b^ (%)	27.33 (43.23)	14.18 (54.41)
Completeness^a^ (%)	90.0 (86.0)	91.0 (90.0)
Redundancy^a^	3.7 (2.7)	5.3 (3.9)
CC_1/2_^a,c^	0.966 (0.880)	0.996 (0.769)
Refinement statistics		
Resolution (Å)	2.27	2.06
*R*_work_ (%)/*R*_free_ (%)^d^	21.20/27.80	16.79/21.29
RMSD		
Bonds (Å)	0.009	0.007
Angles (°)	1.08	0.90
Mean B-factor (Å^2^)	27.48	26.01
Protein	27.35	24.99
Peptide		44.61
Solvent	31.90	32.07
Ramachandran plot (%)^e^	82.2/17.1/0.7/0	85.7/14.0/0.3/0

RMSD, root mean square deviation.

^a^Values in parentheses are for the highest resolution shell.

^b^*R*_merge_ = Σ_h_Σ_i_|*I*_*h*_,_*i*_ − *I*_*h*_|/Σ_h_Σ_i_*I*_*h*_,_*i*_, where *I*_*h*_ is the mean intensity of the *i* observations of symmetry related reflections of *h*.

^c^CC_1/2_ is a percentage of correlation between intensities from random half-datasets^56^.

^d^*R*_work_/*R*_free_ = Σ|*F*_*obs*_ − *F*_*calc*_|/Σ*F*_*obs*_, where *F*_*calc*_ is the calculated protein structure factor from the atomic model (*R*_free_ was calculated with 5% of the reflections selected).

^e^Percentage of residues in most favoured/additionally allowed/generously allowed/disallowed regions of Ramanchandran plot, according to PROCHECK^50^.

**Figure 2:**
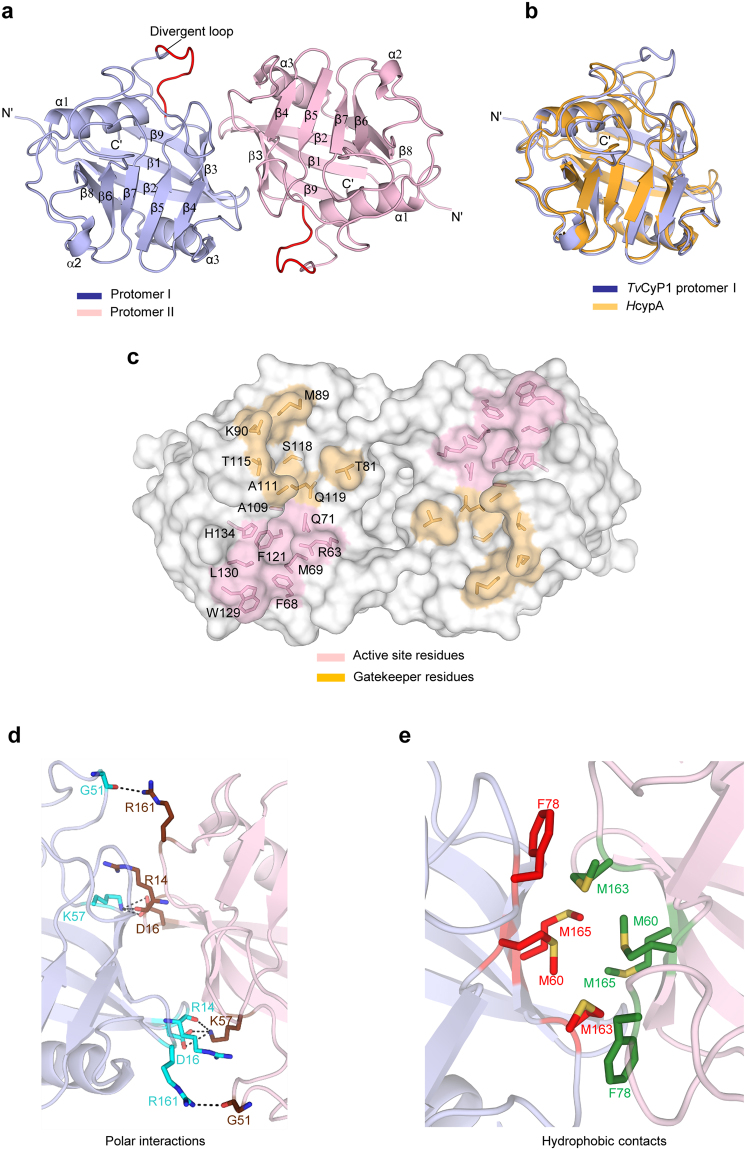
Dimeric structure of *Tv*CyP1 by x-ray crystallography. (**a**) Structure of *Tv*CyP1 dimer showing secondary structural elements (PDB: 5YB9). The divergent loop of *Tv*CyP1 is highlighted in red. (**b**) Structural alignment of *Tv*CyP1 protomer (blue) with *H*cypA (orange). Both structures show the typical cyclophilin fold and exhibit high similarity. (**c**) Surface representation of *Tv*CyP1 highlighting the active site pocket and S2 pocket. The active site residues and “gatekeeper” residues that guard the S2 pocket are shown in sticks. The structure shows the antiparallel nature of *Tv*CyP1 homodimer. (**d** and **e**) Close-up views of polar/salt bridge (**d**) and hydrophobic interactions (**e**) at *Tv*CyP1 dimer interface. The residues that form a dimer interface from either of the protomers are shown in different colored sticks. Dotted lines indicate hydrogen bond/salt bridge interactions. Oxygen, nitrogen and sulfur atoms are shown in red, blue and yellow, respectively.

The crystal structure of *Tv*CyP1 displays the canonical cyclophilin fold with a β-barrel structure composed of eight anti-parallel β-strands capped by two α-helices at the top and bottom (Fig. 2a). With one of the crystallographic symmetry mates, *Tv*CyP1 seems to form an anti-parallel side-to-side dimer, which was earlier confirmed in solution. An interesting structural feature of *Tv*CyP1 is the presence of an extra small beta sheet between α1 and β4. The prototypical cyclophilin, *H*cypA, shows high sequence identity (62%) to *Tv*CyP1. Superposition of *H*cypA (PDB: 1OCA)^31^ with *Tv*CyP1 revealed high structural identity (root mean square deviation [RMSD] = 0.910 Å) between *H*cypA and the protomer of *Tv*CyP1 (Fig. 2b). The active site pocket is far from the dimer interface so that substrate binding to the active site of each protomer is not hindered (Fig. 2c). The dimer interface forms a pocket and is stabilized by hydrogen bonds, salt bridges and hydrophobic interactions between non-conserved residues residing in the loops between β1 and β2, α1 and β3 and α3 and β8. Hydrogen bond interactions between conserved G51 of the divergent loop and R161, R14 and conserved K57 and a salt bridge interaction between D16 and K57 are seen at the dimer interface (Fig. 2d). Hydrophobic interactions at the interface are formed by the residues M60, F78, M163 and M165 (Fig. 2e). Like *C*cyp3, *Tv*CyP1 does not form a disulfide bridge between conserved residues C169 and C41, and these cysteines could play a role in a signaling mechanism during oxidative stress conditions^30^.

### Structure of *Tv*CyP1–Myb1^104–111^ complex

**Figure 3:**
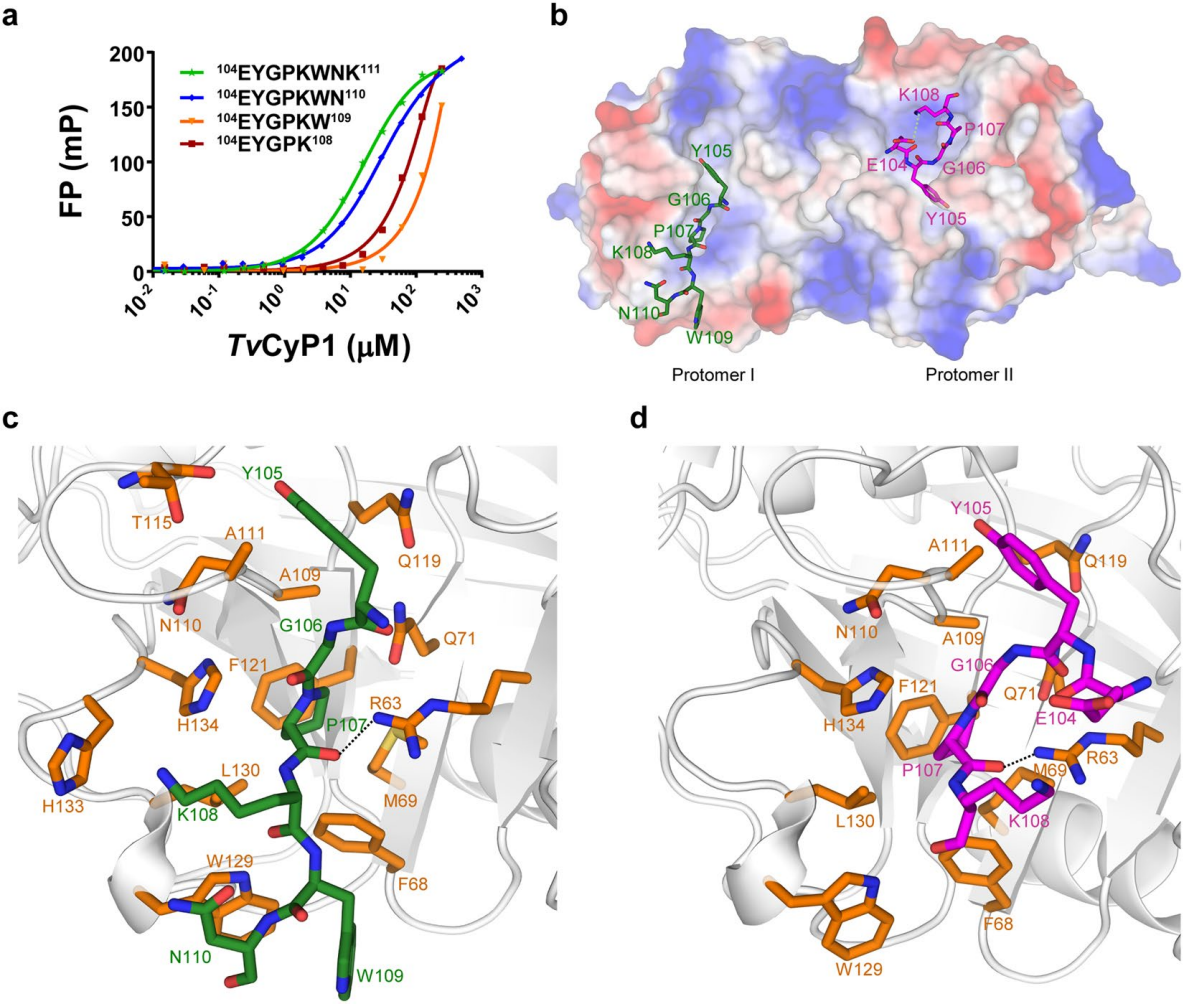
The minimum binding sequence in Myb1 and the complex structure of *Tv*CyP1 with the same. (**a**) Binding curves from fluorescence polarization experiments of Myb1 fragments with *Tv*CyP1. Error bars are standard deviation (SD). Binding affinities were derived from a one-site binding model by using GraphPad Prism 6. ^104^EYGPKWNK^111^ (Myb1^104–111^) showed the highest binding affinity to *Tv*CyP1 (16.81 ± 0.58 μM). (**b**) Electrostatic surface of *Tv*CyP1 dimer in complex with the minimum binding sequence of Myb1, Myb1^104–111^ (shown as green and magenta sticks; PDB: 5YBA). Each protomer binds to one molecule of Myb1 peptide. Although the peptide used was Myb1^104–111^, electron densities of only ^105^YGPKWN^110^ (protomer I) and ^104^EYGPK^108^ (protomer II) were obtained in the complex structure. Intramolecular salt bridge interaction between K108 and E104 is shown as a green dashed line (in ^104^EYGPK^108^ bound to protomer II). (**c** and **d**) Close-up views of interactions between *Tv*CyP1 and Myb1 peptide. Residues of *Tv*CyP1 interacting with Myb1 peptide are shown as orange sticks. Fragments of Myb1 peptide, ^105^YGPKWN^110^ and ^104^EYGPK^108^, bound to either protomer, are represented as green and magenta sticks in (**c**) and (**d**), respectively. The hydrogen bond between the C=O of P107 and the side chain NH_2_ of the catalytic residue R63 is shown as a black dashed line. See Supplementary Fig. S2 for a close-up view of the conformation of the Myb1 peptide bound to either protomer and the conformation of the same sequence in Myb1^35–141^. See Supplementary Fig. S3 also for a 2D diagram by Ligplot+ showing detailed interactions between Myb1 peptide and *Tv*CyP1.

We reported earlier that Myb1 has a minimum *Tv*CyP1 binding sequence, Myb1^104–111^, with ^105^YGP^107^ critical for *Tv*CyP1 interaction^27^. To further confirm this observation, we designed four different fluorescein isothiocyante (FITC)-labeled peptides starting from Myb1^104–108^ (^104^EYGPK^108^) to Myb1^104–111^ (^104^EYGPKWNK^111^) for fluorescence polarization (FP) experiments. Binding isotherms from FP experiments confirmed the eight-residue Myb1 peptide, Myb1^104–111^, having the strongest binding to *Tv*CyP1, with K_d_ value of 16.81 ± 0.58 μM (Fig. 3a).

To obtain a greater clarity on the interaction between *Tv*CyP1 and Myb1^104–111^, we co-crystallized them. The orthorhombic crystals gave optimal-quality X-ray diffraction and belonged to the space group *P*2_1_2_1_2_1_, with the following unit cell dimensions: a = 37.79 Å, b = 78.07 Å, c = 117.71 Å, and α = β = γ = 90°. The structure was solved and refined to 2.06 Å resolution with R_work_ and R_free_ values of 16.79% and 21.29%, respectively (Table 1). The asymmetric unit contained two *Tv*CyP1 molecules forming an anti-parallel side-to-side dimer with non-crystallographic symmetry.

In the initial refinement cycle, continuous electron densities (at 1σ cutoff) located in the active site of *Tv*CyP1 were observed and identified as parts of the Myb1^104–111^ peptide: ^105^YGPKWN^110^ in one protein molecule and ^104^EYGPK^108^ in the other (Fig. 3b). The ^106^GP^107^ atoms were inserted deeply into the hydrophobic active site pocket of *Tv*CyP1, whereas Y105 nestled in the S2 pocket. The complex is formed by several hydrophobic and hydrogen bond interactions of Myb1^104–111^ peptide with the active site and “gatekeeper residues” (Fig. 3c,d and Supplementary Fig. S3). In the active sites of both protomers, *Tv*CyP1 anchors the oxygen of P107 to the hydrophobic pocket by means of hydrogen bond with the guanidinium of its catalytic residue, R63. The overall structures of *Tv*CyP1 in the absence and presence of Myb1^104–111^ were similar, with RMSD 0.225 Å for the main chain. Both structures presented good overall stereochemistry (Table 1) with small RMSD, which suggests that the presence of Myb1^104–111^ did not greatly alter the protein conformation and thus the crystal packing contacts. Presence of Y105 preceding G106 in Myb1^104–111^ enabled optimal interaction with the S2 pocket in *Tv*CyP1.

Myb1^104–111^ peptide bound to both protomers of *Tv*CyP1 showed a difference in the conformation because of different side-chain orientations of K108. In protomer I, ^105^YGPKWN^110^ was in an open conformation, while in protomer II, ^104^EYGPK^108^ adopted a closed conformation due to an intramolecular salt bridge interaction between E104 and K108 (Fig. 3b). However, the two Myb1^104–111^ peptides bound to each protomer adopted a similar conformation of the critical interacting residues ^105^YGP^107^.

### Peptidyl-prolyl isomerase activity of *Tv*CyP1

**Figure 4:**
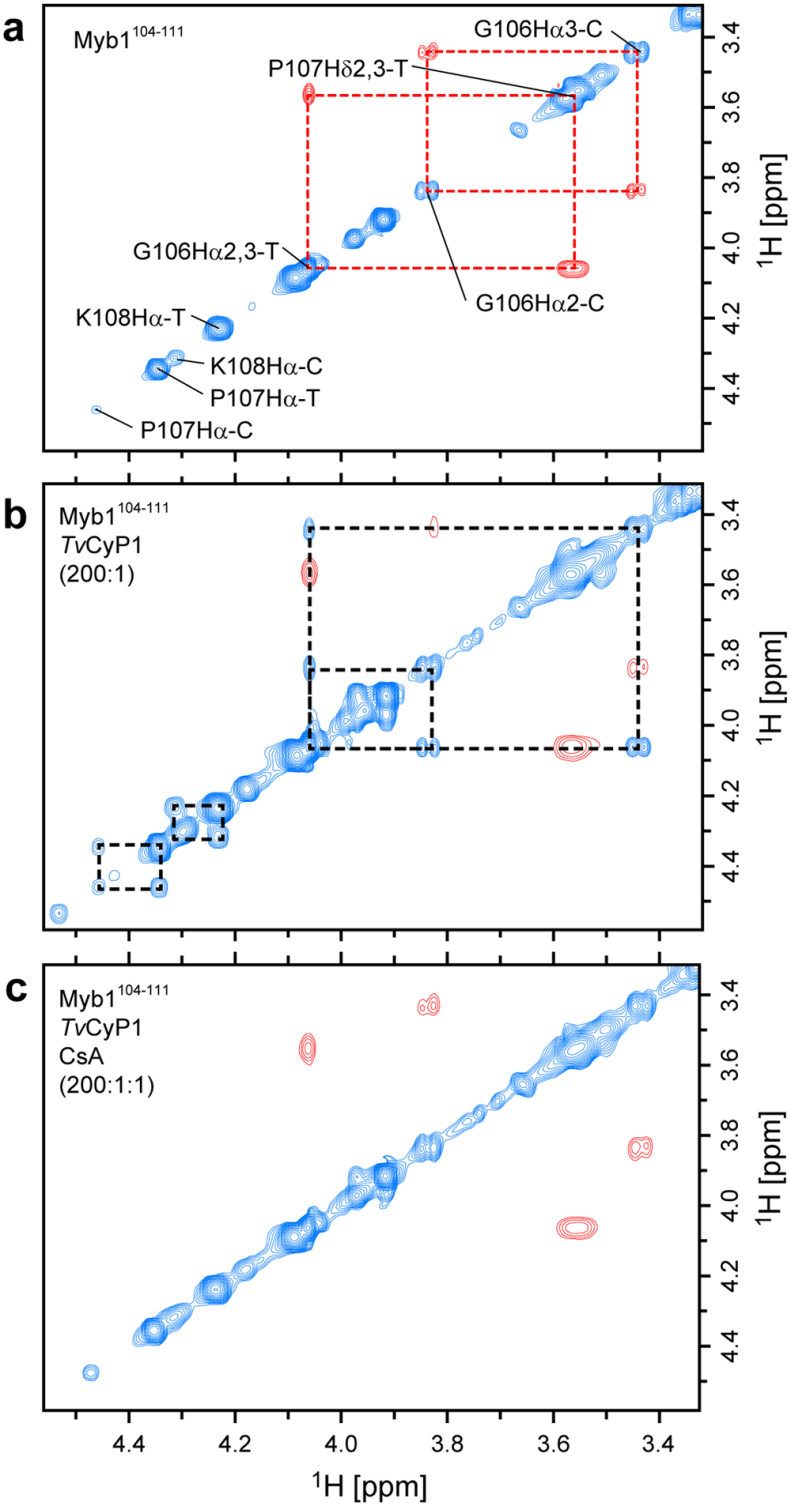
Catalysis of the *cis/trans* isomerization of Myb1^104–111^ peptide by *Tv*CyP1. (**a**) Selected region of the 300-ms mixing-time ROESY spectrum of Myb1^104–111^ peptide (4.4 mM). The positive peaks are colored in blue and negative peaks in red. The ^1^H resonances of G106, P107 and K108 are marked, and the resonances from *cis* and *trans* conformers are labeled as C and T respectively. The negative cross-peaks between neighboring hydrogens are shown in red and connected by red dashed lines. (**b**) Exchange between *cis* and *trans* conformations was accelerated in the presence of 22 μM *Tv*CyP1, as evidenced by the appearance of positive exchange cross-peaks between *cis* and *trans* resonances shown in blue and connected by black dashed lines. (**c**) Inhibition of *Tv*CyP1 isomerase activity by CsA (22 μM) resulted in a loss of the exchange cross-peaks between *cis* and *trans* resonances.

Since *Tv*CyP1 is the only known dimeric cyclophilin with the active sites exposed, we tried to obtain direct evidence for catalysis activity by using NMR rotating-frame Overhauser effect spectroscopy (ROESY). In a ROESY spectrum, the sign of the cross-peaks from chemical exchange is the same as the diagonal peaks, while the cross-peaks between neighboring hydrogens display an opposite sign. In order to monitor the catalysis of *cis/trans* isomerization of P107 in Myb1^104–111^ by *Tv*CyP1, we completed the ^1^H resonance assignments of Myb1^104–111^ peptide. Separate resonance signals were observed for the *cis* and *trans* conformations of residues from Y103 to K111 (Supplementary Fig. S4), indicating that each conformation lies in a distinct chemical environment and the *cis/trans* isomerization is slow on NMR time scale (exchange rate <0.1 s^−1^). Because of the slow exchange rate of the un-catalyzed isomerization, only the cross-peaks between neighboring hydrogens (with opposite sign relative to the diagonal peaks) are observed in the ROESY spectrum (Fig. 4a). Presence of catalytic amounts of *Tv*CyP1 enhanced the *cis/trans* isomerization of P107, as revealed by the appearance of exchange cross-peaks connecting *cis* and *trans* Hα peaks of G106, P107 and K108 (Fig. 4b). The addition of 1.0 molar equivalent of CsA, a strong binding *H*cypA inhibitor, hindered catalysis, as observed by the disappearance of exchange cross-peaks (Fig. 4c). This finding suggests that CsA or CsA analogues can bind to the active site of *Tv*CyP1 with greater affinity than Myb1^104–111^ and hence can be used to inhibit the *Tv*CyP1–Myb1 interaction. All these data provide a direct evidence for the catalysis of *cis/trans* isomerization of P107 in Myb1^104–111^ peptide by *Tv*CyP1.

### Identification of *Tv*CyP1-binding site on Myb1^35–141^ protein

**Figure 5:**
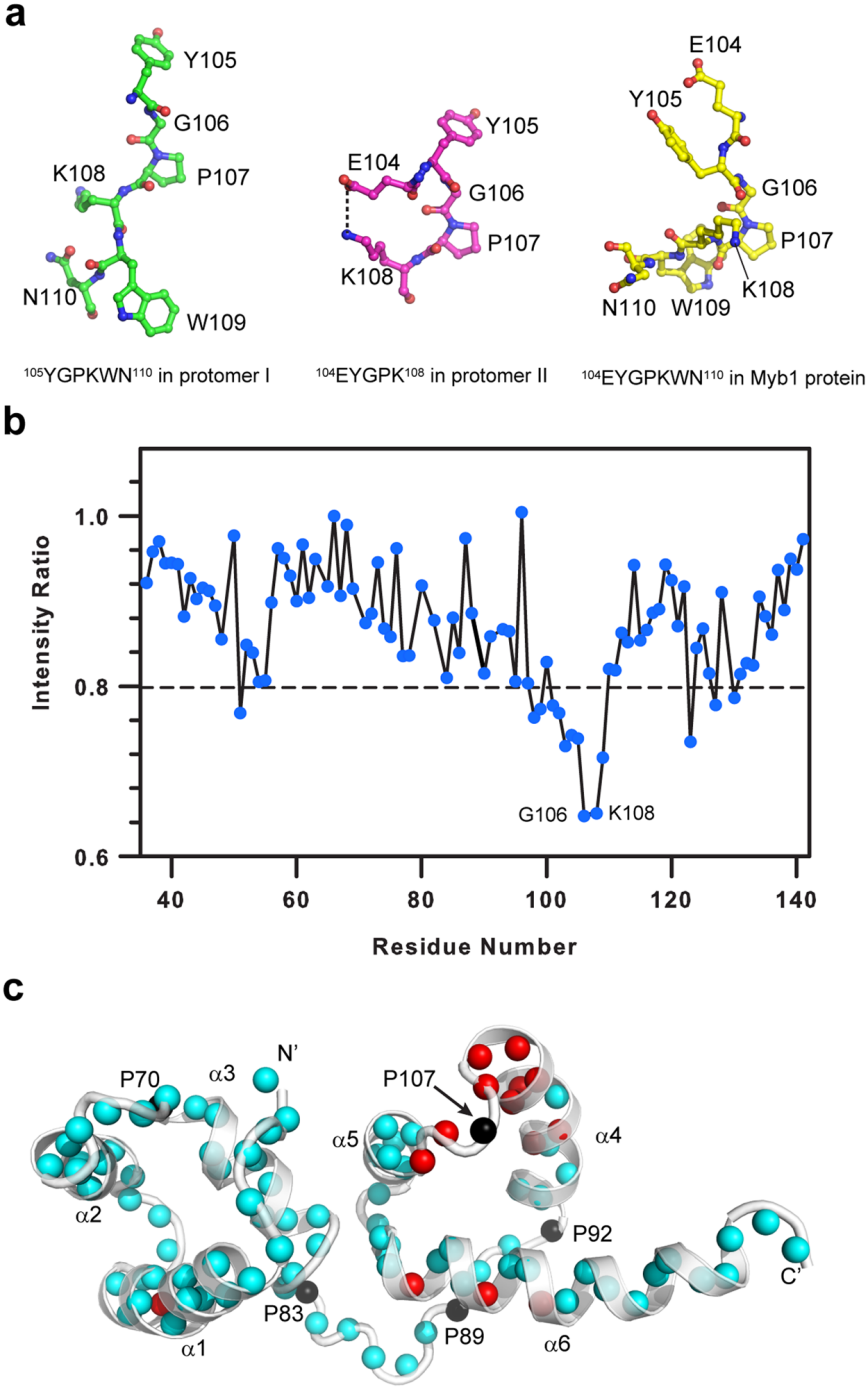
*Tv*CyP1 interacts with the P107-containing loop in Myb1^35–141^ protein. (**a**) Conformations of the minimum binding sequence in protomer I (left), protomer II (middle) and Myb1^35–141^ protein (right), showing that the structure of this sequence in 3 states is different from each other. (**b**) The intensity ratio of the amide proton resonances, with and without irradiation of the aliphatic protons, plotted against the Myb1^35–141^ residue number. The dashed line signifies one SD from the average intensity ratio (~0.8). (**c**) Mapping the cross-saturation transfer NMR results on Myb1^35–141^ protein structure. Proline residues are shown as black spheres. The residues exhibiting intensity ratios lower that 0.8 are shown as red spheres and others as cyan spheres. Since *Tv*CyP1 is a PPIase, significant reduction in peak intensities of residues near P107 indicates that *Tv*CyP1 binds to the loop containing P107.

Unlike the typical substrates for cyclophilins that exhibit flexible structures to fit into the active site pocket, Myb1 is a well-folded protein with *Tv*CyP1-binding key residues, ^105^YGP^107^, lying in a short loop structurally restrained by two stable helices. Hence, the conformation of the minimum binding sequence, ^104^EYGPKWNK^111^, in Myb1 protein is very different from those observed in the complex structure (Fig. 5a). Our previous NMR structural study on Myb1^35–141^ indicated that all residues from E104 to K111 adopt a *trans* conformation and there was no NMR signal from the *cis* conformer^32^. As a result, it was not possible to study the catalysis of *cis/trans* isomerization of ^106^GP^107^ bond in Myb1^35–141^ protein by *Tv*CyP1. Also, we tried to co-crystallize the *Tv*CyP1–Myb1^35–141^ protein complex but could not obtain any crystal.

To identify the *Tv*CyP1-binding site on Myb1^35–141^ protein, we used NMR. The titration of unlabeled *Tv*CyP1 to ^15^N-labeled Myb1^35–141^, which includes the R2-R3 DNA binding domains of Myb1, resulted in significant line-width broadening of all residues but very little chemical shifts perturbation (Supplementary Fig. S5). To identify the *Tv*CyP1 binding site on Myb1^35–141^ protein, we used cross-saturation transfer NMR that helps to identify the residues involved in direct intermolecular contacts^33^. We prepared uniformly ^15^N, ^2^H (>95%)-labeled Myb1^35–141^ to avoid excitation of its aliphatic protons by the radio-frequency pulses. The labeled Myb1^35–141^ protein mixed with unlabeled *Tv*CyP1 was dissolved in 50% ^2^H_2_O to reduce saturation transfer from spatially crowded amide protons by ^1^H_2_O. The plot of intensity ratios of amide peaks with and without saturation versus residue number showed a significant decrease in amide peak intensity for the residues near P107, with the minimum at G106 and K108 (Fig. 5b). Although there are 5 proline residues in Myb1^35–141^, P70, P83, P89, P92 and P107, cross-saturation transfer NMR clearly showed that *Tv*CyP1 interacts with the P107-containing loop in Myb1^35–141^ protein (Fig. 5c).

### Mapping the Myb1^35–141^ protein interaction region on *Tv*CyP1

**Figure 6:**
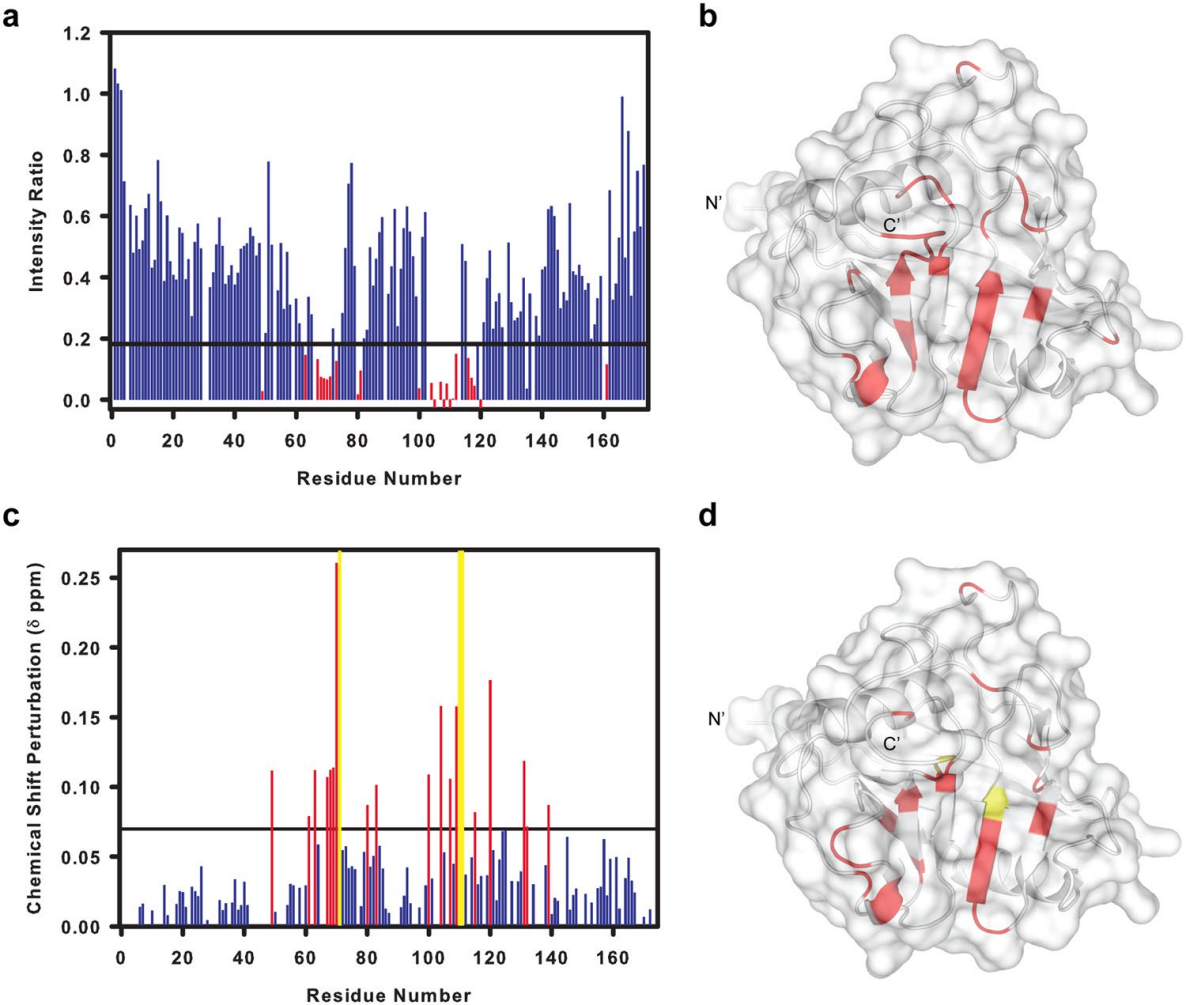
Interaction of *Tv*CyP1 with Myb1^35–141^ protein is similar to that of its binding to Myb1^104–111^peptide. (**a**) Plot of the ratio of intensities of *Tv*CyP1 peaks in the presence and absence of Myb1^35–141^ (1:2) against the residue number. Gaps indicate unassigned or proline residues. Red bars indicate the intensity ratio less than the lower control line (Average − SD). Residues that disappeared completely are shown as negative red bars. See Supplementary Fig. S6 for titration HSQC overlay. See also Supplementary Fig. S7 for the interaction between *Tv*CyP1 and Myb1^35–141^ mutant P107A. (**b**) Cartoon structure of *Tv*CyP1 protomer showing line-width–broadened or disappeared residues on titration of Myb1^35–141^ highlighted in red. (**c**) The CSP values for backbone amide resonances of *Tv*CyP1 on titration with Myb1^104–111^ (1:2). Red bars indicate CSP values more than the upper control line (Average + SD) and yellow bars indicate backbone amide resonances that disappeared on titration. See Supplementary Fig. S6 for titration HSQC overlay. (**d**) Cartoon structure of *Tv*CyP1 protomer showing chemical shift-perturbed and disappeared residues on titration of Myb1^104–111^ highlighted in red and yellow, respectively.

Further, we tried to map the Myb1^35–141^ protein binding site on *Tv*CyP1 by NMR. To achieve this goal, we firstly completed the ^1^H, ^13^C and ^15^N backbone chemical shift assignment of *Tv*CyP1 using standard triple-resonance NMR spectra acquired from a ^2^H, ^13^C, ^15^N (>95%)-labeled protein sample. The ^1^H, ^15^N TROSY-HSQC of *Tv*CyP1 features an extremely well dispersed set of resonances (Supplementary Fig. S2), agreeing with the well-folded structure of this protein. To map the Myb1^35–141^ protein interaction site on *Tv*CyP1, we titrated 2 folds of unlabeled Myb1^35–141^ protein to ^15^N-labeled *Tv*CyP1, resulting in severe line-width broadening of the peaks of the active site and “gatekeeper” residues of *Tv*CyP1 (Fig. 6a,b and Supplementary Fig. S6). Apart from the active site and “gatekeeper” residues, certain residues in faraway loops were also perturbed or line-width–broadened during titration owing to conformational exchange during substrate turnover^34^. To confirm the specificity of interaction, we produced a Myb1^35–141^ mutant protein with P107 mutated to alanine (P107A-Myb1^35–141^) and titrated unlabeled P107A-Myb1^35–141^ to ^15^N-labeled *Tv*CyP1 (Supplementary Fig. S7). The nearly identical NMR spectra from *Tv*CyP1 with and without P107A- Myb1^35–141^ suggested that *Tv*CyP1 recognizes P107A-Myb1^35–141^ weakly. Of note, despite 5 proline residues in Myb1^35–141^, only P107 can bind to *Tv*CyP1 active site pocket specifically.

Although we tried to measure the binding affinity of *Tv*CyP1–Myb1^35–141^ interaction using several biophysical techniques, we were not successful. Hence, to compare *Tv*CyP1–Myb1^104–111^ and *Tv*CyP1–Myb1^35–141^ interactions, we also titrated Myb1^104–111^ to ^15^N-labeled *Tv*CyP1. The titration resulted in a notable chemical shift perturbation (CSP) of several *Tv*CyP1 residues (Fig. 6c and Supplementary Fig. S6). Structural mapping of these residues showed that most of the *Tv*CyP1 residues that underwent significant chemical shift perturbation on Myb1^104–111^ peptide titration also exhibited severe line-width broadening on Myb1^35–141^ protein titration (Fig. 6d). Although, in the well-folded Myb1^35–141^ protein, the minimum binding sequence adopts a very different conformation from that observed in the complex structure, our NMR studies suggest that the interaction between *Tv*CyP1 and Myb1^35–141^ protein is similar to the highly specific recognition between *Tv*CyP1 and Myb1^104–111^ peptide.

### Interaction with *Tv*CyP1 reduces the slow dynamics in Myb1^35–141^ around P107

**Figure 7:**
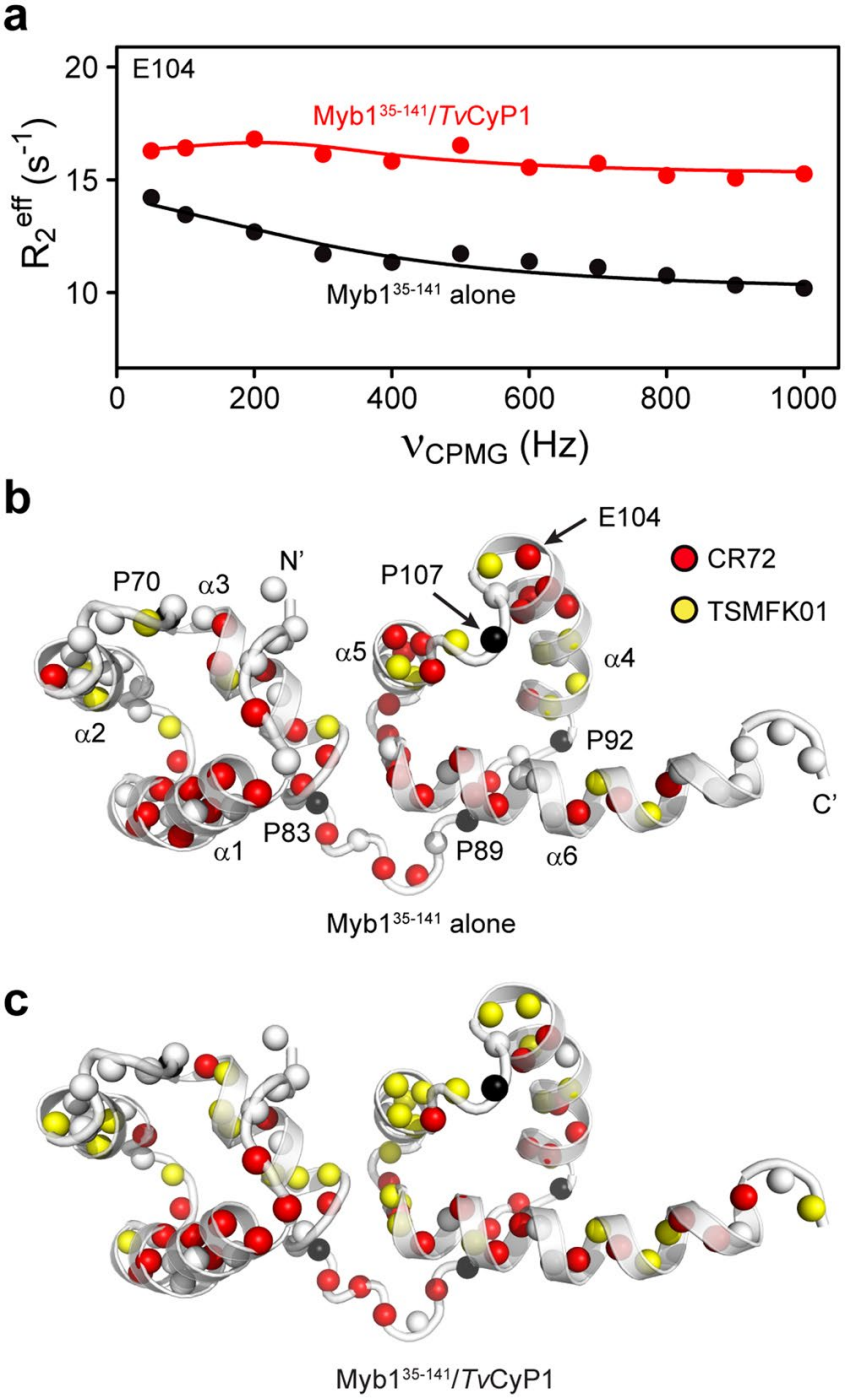
Myb1 dynamics is reduced by the binding of *Tv*CyP1. (**a**) Relaxation dispersion data for E104 of Myb1^35–141^ in the free form (black circles) and in complex with *Tv*CyP1 (red circles) measured at 850 MHz. Lines show individual fits to a two-site exchange process, demonstrating very slow exchange fit with the TSMFK model or faster exchange fit with the CR72 model, corresponding to yellow and red spheres, respectively, in the structural map in (**b**) and (**c**). Black spheres denote proline residues not observed by ^1^H,^15^N HSQC NMR and white spheres indicate overlapped residues that could not be analyzed or the residues that do not show slow dynamics. (**b**) Mapping the models of dispersion data of free Myb1^35–141^, showing that a lot of residues in Myb1 have faster conformational exchange fit with the CR72 model. (**c**) In the presence of *Tv*CyP1, the dynamics of E104 and several residues in α5 are restricted to become very slow dynamics and can be fitted with the TSMFK model.

We carried out NMR Carr-Purcell-Meiboom-Gill (CPMG) relaxation dispersion experiments at ^1^H frequency of 600 and 850 MHz on Myb1^35–141^ in the free form and in complex with *Tv*CyP1 to probe the millisecond time scale conformational exchange of Myb1^35–141^ in 2 different states. The ^15^N relaxation dispersion profiles of individual residues of Myb1^35–141^ were fitted to a two-site exchange process using CR72^35^ or TSMFK01^36^ model by the software relax^37^ (Fig. 7a) and the extracted dynamic parameters are listed in Supplementary Tables S1 and S2 for the CR72 and TSMFK01 models, respectively. Plenty of residues in free Myb1^35–141^ displayed conformational exchange (Fig. 7b), suggesting that Myb1 is a highly dynamic protein. However, the exchange rates for the residues around P107 are too different to be fitted globally to a single model. The residues with very slow exchange rates were fitted to the TSMFK01 model and those with faster exchange rates were fitted to the CR72 model, shown as yellow and red spheres respectively in Fig. 7b,c. In free Myb1^35–141^, E104 and several residues in α5 exhibit faster dynamic motions (Fig. 7b). And most of these motions are restricted to become very slow dynamics by *Tv*CyP1 binding (Fig. 7c). Together, NMR CPMG relaxation dispersion data show that the highly dynamic motions of the Myb1 residues around P107 are significantly restricted on interaction with *Tv*CyP1.

## Discussion

Dimerization via the interaction of hydrophobic residue pairs in a mirror-image–like fashion as seen in *Tv*CyP1 has been observed in various dimeric bacterial FKBP-type PPIases^38,39^, but not in any other cyclophilins. Most of the cyclophilins known to date are monomeric in nature, with certain exceptions such as *Aspergillus fumigatus* cyclophilin (Asp f11; PDB: 2C3B), cyclophilin from Mimivirus (Mimicyp; PDB: 2OSE) and cyclophilin from *Hirschia baltica* (AquaCyp300; PDB: 5EX1). However, AquaCyp300 is not a single domain cyclophilin because N- and C-terminal extensions together with the large insertion in the cyclophilin domain form a contiguous structural entity termed the NIC domain^40^. The dimer interface in AquaCyp300 is formed by residues of both the cyclophilin domain and NIC domain. Although Asp f11 and Mimicyp are single-domain cyclophilins with swapped dimeric and trimeric structures, respectively, they were found to be monomeric in solution^28,29^. Moreover, multimerization in these two proteins resulted in obstruction of the active site, thereby hindering substrate interaction. In contrast, *Tv*CyP1 exhibits a natural tendency to form a dimer even in solution, as observed from a single dimeric peak in SEC-MALS and AUC-SV analyses, and has the active site of both protomers exposed for interaction with the substrate. Hence, *Tv*CyP1 is the only single-domain dimeric cyclophilin reported so far that can still bind to its substrates.

The complex structures of cyclophilin with its biologically relevant substrate are available for only *H*cypA. The complex structures of *H*cypA with HIV capsid protein and CrkII protein show high structural similarity because both contain a Gly-Pro motif binding to the active site with proline fitting deep inside the hydrophobic pocket of the active site^41,42^. However, neither of these biological substrates show strong interaction with the S2 pocket because of the absence of residues that exhibit surface complementarity to the S2 pocket of *H*cypA.

Myb1 is an ideal cyclophilin substrate due to the presence of Tyr-Gly sequence preceding the interacting proline that enables a strong interaction with the active site and S2 pocket of *Tv*CyP1^6,43^. As a result, in the *Tv*CyP1–Myb1^104–111^ complex structure, we observed that Y105 was inserted well inside the S2 pocket. However, Myb1^35–141^ is a well-folded protein with interacting proline, lying in a short loop surrounded by two stable helices. Hence, in the well-folded Myb1 protein, the interaction of Y105 with the S2 pocket is structurally hindered and therefore could occur only if some conformational changes happen at helix 4, where it is located. This was found to be true from CPMG relaxation dispersion experiments which showed that Myb1^35–141^ underwent slow conformational exchange. The slow dynamics near the region containing P107 was greatly reduced upon interaction with *Tv*CyP1 further suggesting that the interaction is facilitated by dynamics.

In the complex structure, we observed a difference in the conformation of Myb1^104–111^ peptide bound to both protomers of *Tv*CyP1 because of different side-chain orientations of K108. Structural comparison showed that the conformation of ^104^EYGPK^108^ in protomer II is more similar to the conformation of the loop in Myb1 containing P107 (Fig. 5a). Hence, with some conformational changes at Y105, the minimum binding sequence in Myb1^35–141^ upon interaction with *Tv*CyP1 could probably adopt a similar structure as that of ^104^EYGPK^108^, bound to protomer II.

Nuclear translocation of Myb1 is an important step regulating the expression of disease causing *ap65–1* gene. Hence, preventing the nuclear import by blocking the interaction between Myb1 and *Tv*CyP1 can be a good strategy to treat the disease. From ROESY experiments, we observed that CsA can inhibit Myb1^104–111^ binding to the active site. Although CsA has long been proposed to be an inhibitor for cyclophilin^4^, the deleterious effect of its immunosuppressive activity^44^ has always been a major hindrance in using it as a drug to treat diseases. Moreover, CsA is not specific to any of the isoforms of cyclophilins^6^. Many attempts to design and synthesize an isoform-specific drug for cyclophilins have not been successful and the S2 region has been proposed as an ideal site for isoform-specific drug development^6^. However, because *H*cypA and *Tv*CyP1 possess the same set of “gatekeeper” residues, a drug targeting the S2 region of *Tv*CyP1 may also be able to interact with *H*cypA in humans. However, the harmful effect of this unwanted interaction would probably not be as detrimental as immunosuppression and hence is worth studying. An alternative and probably better drug target site would be the dimer interface. As shown in Fig. 2c, there is a pocket formed by the dimer interface in *Tv*CyP1. A CsA analogue that can recognize both the active site and the dimer interface pockets may specifically recognize *Tv*CyP1 but not the monomeric *H*cypA.

Collectively, our study has helped in understanding the unique dimeric structure of *Tv*CyP1 and obtaining detailed information about its interaction with the biological substrate Myb1. *Tv*CyP1 has also been found to interact with Myb3^27^, and future work will elucidate the details of the Myb3–*Tv*CyP1 interaction. It is also possible that *Tv*CyP1 dimerization may help in the simultaneous binding of Myb1 and Myb3 at the active sites of either protomer. Indeed, much needs to be studied at the cellular level to further understand the role of *Tv*CyP1 and the significance of its dimerization on the parasite’s development and pathogenicity. However, our current data shows that *Tv*CyP1 is a good drug target and provides a rationale to design drugs specific for the protein or to disrupt its interaction with Myb1.

## Methods

### Expression and purification of *Tv*CyP1 and *Tv*CyP1 mutants

*Tv*CyP1 gene was cloned into pET-28a vector (Novagen) and was expressed with an N-terminal His-tag followed by a TEV digestion recognition site in the *Escherichia coli* strain BL21(DE3). After TEV protease digestion, *Tv*CyP1 protein had an additional Glycine and Serine at its N-terminus. For unlabeled or uniformly labeled samples, *E. coli* cells were cultured in lysogeny broth medium or M9 minimal medium at 37 °C, respectively. The recombinant *Tv*CyP1 was expressed at 16 °C by the addition of 0.5 mM isopropyl β-D-1-thiogalactopyranoside (IPTG) with the culture medium reaching optical density at 600 nm of 0.6. Cells were harvested 16 hr after induction and resuspended in Tris-HCl (pH 8.0) and lysed by using a M-110S microfluidizer (Microfluidics). The insoluble fraction was removed by centrifugation at 12,000 rpm for 30 min. The supernatant was passed through Q ion-exchange resin (Q sepharose fastflow, GE healthcare) and the flow-through containing *Tv*CyP1 was further purified by nickel-nitrilotriacetic acid (Ni-NTA) affinity resin (Qiagen, Hilden, Germany) equilibrated with 20 mM Tris-HCl, 100 mM NaCl, and 10 mM Imidazole (pH 8.0). *Tv*CyP1 was eluted from the Ni-NTA resin in 500 mM imidazole, 100 mM NaCl and 20 mM Tris solution at pH 8.0. The eluate was treated with TEV protease overnight at room temperature and then diluted to a final imidazole concentration of 10 mM and passed through the Ni-NTA resin again. The flow-through containing *Tv*CyP1 was buffer-exchanged in 20 mM NaH_2_PO_4_, 50 mM NaCl and 0.5 mM NaN_3_ (pH 6.0) by centrifugation with 10,000 Da MWC membrane ultrafiltration (Millipore). The ^2^H/^13^C/^15^N-labeled *Tv*CyP1 was prepared in the same way, but the host cells were grown in M9 minimal medium containing D_2_O supplemented with ^15^N-NH_4_Cl (1 g/L) and ^13^C-glucose (2 g/L). The protein purity was verified by SDS-PAGE, and the concentration of *Tv*CyP1 monomers was assessed by using a molar absorption coefficient of E^280^ = 8,480 M^−1^. *Tv*CyP1 mutants were expressed and purified using the same protocol.

### Preparation of Myb1, Myb1 mutants and Myb1 peptides

The gene encoding Myb1^35–141^ was cloned into the pET-29b (Novagen) vector and expressed in *E. coli* BL21(DE3) cultured in lysogeny medium or minimal medium for ^1^H/^15^N-labeled samples. The expressed Myb1^35–141^ has two extra residues in the C-terminus, leucine and glutamic acid, followed by a His-tag. Myb1^35–141^ was expressed and purified as described^32^. ^2^H,^13^C,^15^N-labeled Myb1 was prepared in the same way, but the host cells were grown in M9 minimal medium containing D_2_O supplemented with ^15^NH_4_Cl (1 g/L) and ^13^C, ^2^H glucose (2 g/L). The purity of Myb1^35–141^ was confirmed by SDS-PAGE and the concentration was calculated by using a molar absorption coefficient of E^280^ = 33,460 M^−1^. The final protein sample used for experiments was in a buffer containing 20 mM NaH_2_PO_4_, 50 mM NaCl and 0.5 mM NaN_3_ (pH 6.0). Myb1 mutants were expressed and purified using the same protocol. Myb1 peptides were purchased from Yao-Hong Biotechnology Inc. (Taiwan) and FITC-labeled Myb1 peptides were synthesized by the peptide synthesis facility at the Institute of Biological Chemistry, Academia Sinica.

### Size-exclusion chromatography coupled to multi-angle static light scattering (SEC-MALS)

A total of 300 μg of *Tv*CyP1 was injected into a size-exclusion chromatography column (ENrich™ SEC. 70 10 × 300 Column, Bio-Rad Laboratories, Inc.) and analyzed by static and dynamic light scattering^45^ at a flow rate of 0.5 mL min^−1^ in 20 mM monosodium phosphate, 50 mM NaCl, 0.5 mM NaN_3_ (pH 6.0) at 25 °C. The column is in line with four detectors: a static light-scattering detector (miniDAWN TREOS, Wyatt Technology), a quasi-elastic light-scattering detector (QELS, Wyatt Technology), a refractive index detector (Optilab T-rEX, Wyatt Technology) and an ultraviolet-visible (UV) detector (Agilent, USA). Bovine serum albumin (Sigma, A1900) was used for system calibration. Molecular weight was calculated by using ASTRA 6 (Wyatt Technology) with the dn/dc value set to 0.185 mL g^−1^.

### Analytical ultracentrifugation

AUC-SV experiments were conducted on a Beckman Coulter ProteomeLab XL-I ultracentrifuge with an AN 50Ti rotor. The sample was collected from a Superdex 75 gel filtration column (GE Healthcare Life Sciences) after equilibrating the column extensively with 20 mM monosodium phosphate, 50 mM NaCl, 0.5 mM NaN_3_ (pH 6.0). We loaded 400 μl of 29 μM *Tv*CyP1 and collected data at 20 °C at 50,000 rpm at 280 nm. The scans were collected every minute, with a total 150 scans obtained per sample. Buffer density, viscosity and partial specific volume were calculated by using SednTerp. The data were edited and processed by using SedFit software (https://sedfitsedphat.nibib.nih.gov).

### Fluorescence polarization measurements

Myb1 peptides for fluorescence polarization experiments were labeled with FITC at the N-terminus. The indicated amounts of *Tv*CyP1 were added to wells containing 0.5 μM FITC-labeled Myb1 peptide in 20 mM monosodium phosphate, 50 mM NaCl, 0.5 mM NaN_3_ (pH 6.0) at 298 K. Triplicate measurements of the reactions were acquired by using the SpectraMax Paradigm microplate reader (Molecular Devices, CA, USA) with excitation wavelength 485 nm and emission wavelength 535 nm. Data were analyzed and fitted to a one-site binding model by using GraphPad Prism 6 (San Diego, CA, USA).

### Protein crystallization and data collection

For crystallization screening experiments, *Tv*CyP1 sample was further purified on a Superdex 75-gel filtration column (GE Healthcare Life Sciences) after equilibrating the column extensively with 20 mM Bis-Tris, 50 mM NaCl and 0.5 mM NaN_3_ (pH 6.0). Initial protein crystallization trials were performed at 283 K by the sitting-drop vapour-diffusion method with commercial crystallization screen kits, 96-well Intelli-plates (Art Robbins Instruments) and a Phoenix robot (Art Robbins Instruments). For co-crystallizing with Myb1^104–111^ peptide, purified *Tv*CyP1 protein was mixed with the synthetic Myb1^104–111^ peptide (Glu-Tyr-Gly-Pro-Lys-Trp-Asn-Lys) in a molar ratio of 1:4 before crystallization trials. Each crystallization drop was prepared by mixing 0.3 μL *Tv*CyP1 or *Tv*CyP1/peptide at 9 mg/mL with an equal volume of mother liquor, and the mixture was equilibrated against 100 μL reservoir solution. The crystals of *Tv*CyP1 in apo and Myb1 peptide-bound forms were grown at 283 K within 7–14 days with the optimal conditions of 100 mM HEPES, pH 7.0, 30% (v/v) Jeffamine M-600 and 100 mM Tris-HCl pH 8.0, 30% (v/v) polyethylene glycol 400, respectively. For diffraction data collection, the crystal was cryoprotected in mother liquor supplemented with 20% glycerol, and flash-frozen in liquid nitrogen at 100 K. The diffraction images of *Tv*CyP1 in apo and Myb1 peptide-bound forms were recorded at the National Synchrotron Radiation Research Center (Taiwan) on a MX300HS detector in TPS 05A and a Q315r detector in TLS BL13C1 beamlines, respectively. Diffraction data were processed and scaled by using HKL2000 software^46^.

### Structure determination and refinement

The crystal structures of *Tv*CyP1 and *Tv*CyP1 in complex with Myb1^104–111^ peptide were determined by molecular replacement with the Phaser-MR program^47^, by using the *C*cyp3 structure from *C. elegans* (PDB: 1DYW)^30^ as a search model. Crystallographic refinement involved repeated cycles of conjugate-gradient energy minimization and temperature-factor refinement with the program phenix.refine in the PHENIX package^48^. Amino-acid side chains and water molecules were fitted into 2Fo-Fc and Fo-Fc electron-density maps by using COOT^49^. The model was evaluated by using PROCHECK^50^ and MOLPROBITY^51^. The data collection and structure refinement statistics are in Table 1. Final coordinates and structure factors of *Tv*CyP1 and the *Tv*CyP1–Myb1 peptide complex have been deposited in the Protein Data Bank (PDB: 5YB9 and 5YBA, respectively).

### NMR experiments

*Tv*CyP1 and Myb1^35–141^ samples for NMR experiments were prepared in 20 mM monosodium phosphate, 50 mM NaCl, 0.5 mM NaN_3_ (pH 6.0). To improve the spectral quality of *Tv*CyP1, the sample used for backbone assignment was ^2^H,^13^C,^15^N-labeled. The NMR spectra were acquired on a Bruker AVANCE 600-, 800- and 850-MHz spectrometers equipped with a z-gradient TXI cryoprobe (Bruker, Karlsruhe, Germany) at 310 K. Backbone assignment of *Tv*CYP1 was based on TROSY-HNCACB and -HNCA spectra^52^. Backbone assignment of FM-*Tv*CyP1 was based on the HNCACB spectrum with a ^13^C, ^15^N-labeled sample. Backbone assignment of Myb1^35–141^ was reported previously^32^. Observation of chemical-shift changes as well as reduction in peak intensity in ^1^H,^15^N TROSY-HSQC of ^15^N-enriched *Tv*CyP1 upon titrating unlabeled Myb1 peptide or Myb1^34–141^ was used to confirm interactions and to determine the binding site of *Tv*CyP1. The weighted CSPs for backbone ^15^N and ^1^H_N_ resonances were calculated with the equation Δδ = [((Δδ_HN_)^2^ + (Δδ_N_/5)^2^)/2]^0.5^.

All experiments with Myb1 were performed at 298 K. The sample for cross-saturation transfer experiments contained a ^2^H, ^13^C, ^15^N-labeled Myb1^35–141^ sample (333 μM) and unlabeled *Tv*CyP1 (1000 μM) in 20 mM monosodium phosphate, 50 mM NaCl, 0.5 mM NaN_3_ (pH 6.0). To avoid spin diffusion, the sample was prepared in 50% D_2_O. The deuteration percentage of Myb1^35–141^ is more than 95%. The saturation pulses were centered at 516.8 Hz (0.608 ppm) at which we observed large proton peaks from *Tv*CyP1 and no peak from Myb1^35–141^. The measurement time was 5.5 h, with relaxation delay and saturation time 5 s.

^1^H-TOCSY, ^1^H-COSY and ^1^H-NOESY were performed at 300 K with a mixing time of 75 ms (TOCSY) and 300 ms (NOESY) on the Myb1^104–111^ peptide to complete the assignment of proton resonances. The peptide was dissolved in 20 mM monosodium phosphate, 50 mM NaCl, 0.5 mM NaN_3_ (pH 6.0) to obtain a concentration of 2 mM. All ROESY experiments were performed at 300 K with 300 ms mixing time. For catalysis experiments, Myb1^104–111^ peptide (2000 μM) was mixed with *Tv*CyP1/FM-*Tv*CyP1 (13.3 μM) at a ratio of 150:1.

The ^15^N, ^2^H-labeled Myb1^35–141^ (0.3 mM) in the free form and in complex with unlabeled *Tv*CyP1 (0.36 mM) or unlabeled FM-*Tv*CyP1 (0.36 mM) were prepared for CPMG measurement. The constant-time ^15^N single-quantum relaxation dispersion experiments^36^ were acquired at 298 K on both 600 MHz and 850 MHz NMR spectrometers with ν_CPMG_ of 50, 100, 200, 300, 400, 500, 600, 700, 800, 900 and 1000 Hz and a total CPMG delay of 40 ms. The spectra with ν_CPMG_ of 100 and 700 Hz were collected twice to estimate experimental errors. The peak heights in each spectrum were extracted by using an automated routine in NMRView. The *R*_2,eff_ was calculated as *R*_2,eff_ (ν_CPMG_) = (−1/T) ln[I(ν_CPMG_)/I0], where I(ν_CPMG_) and I0 are the intensities of peaks recorded with and without the CPMG intervals. The ^15^N relaxation dispersion profiles of individual residues at both 600 and 850 MHz were fitted to a two-site exchange process by the software relax^37^. Two models, the Carver and Richards equation^35^ (CR72) that describes 2-site exchange for most time scales and TSMFK01^36^, which is appropriate for 2-site very slow exchange within range of microsecond to second time scale, were fitted. The Akaike’s model selection^53^ was performed to judge statistical significance of the models. Successful fit to CR72 model yields the population-average transverse relaxation rate (*R*_2_^0^), the exchange rate (*k*_ex_), the population of the major state (*p*A) and the chemical shift difference between 2 states (Δω). The fit to TSMFK01 yields *R*_2A_^0^, *k*_ex_, and Δω. The extracted dynamic parameters are listed in Supplementary Tables S1 and S2 for the CR72 and TSMFK01 models, respectively.

All NMR spectra, except the spectra for cross-saturation transfer and CPMG relaxation dispersion experiments, were processed by using Topspin 3.1 (Bruker) and analyzed by using NMRViewJ^54^. For cross-saturation transfer experiments, the spectra were processed by using Topspin 3.1 (Bruker) and analyzed by using Sparky (T.D. Goddard and D.G. Kneller, Sparky 3, University of California, San Francisco). For CPMG-based relaxation dispersion experiments, the spectra were processed by using NMRpipe^55^ and analyzed by using NMRviewJ.

### Data availability

The datasets generated during the current study are available in the RCSB PDB repository, [https://www.rcsb.org/pdb/home/home.do]. The backbone chemical shift assignment of *Tv*CyP1 generated during the current study are available in BMRB under accession code 12014.

### Accession Numbers

The atomic coordinates and structure factors for *Tv*CyP1 and *Tv*CyP1-Myb1 peptide complex are deposited under RCSB PDB accession codes PDB: 5YB9 and 5YBA respectively. The backbone NMR chemical shift of *Tv*CyP1 has been deposited in the BMRB under accession code 12014.

## Electronic supplementary material

Supplementary Information
