# Supplementary material for: Structural basis of interaction between dimeric cyclophilin 1 and Myb1 transcription factor in Trichomonas vaginalis
Source: Sci Rep. 2018 Apr 3;8:5410. doi: 10.1038/s41598-018-23821-5 (PMC5882848; doi:10.1038/s41598-018-23821-5)
Supplement: Supplementary file 1 — Supplementary Information [file 41598_2018_23821_MOESM1_ESM.pdf]

Supplementary Information for

Structural basis of interaction between dimeric cyclophilin 1 and Myb1 transcription factor in *Trichomonas vaginalis*

Tesmine Martin[1,2,3], Yuan-Chao Lou[1], Chun-Chi Chou[1], Shu-Yi Wei[1], Sushant Sadotra[1,2,4], Chao-Cheng Cho[5], Meng-Hsuan Lin[5], Jung-Hsiang Tai[1], Chun-Hua Hsu[5,6,*] and Chinpan Chen[1,*]

[1]Institute of Biomedical Sciences, Academia Sinica, Taipei 115, Taiwan
[2]Chemical Biology and Molecular Biophysics, Taiwan International Graduate Program, Academia Sinica. Taipei 115, Taiwan
[3]Department of Chemistry, National Tsing Hua University, Hsinchu 300, Taiwan
[4]Institute of Bioinformatics and Structural Biology, National Tsing Hua University, Hsinchu 300, Taiwan
[5]Genome and Systems Biology Degree Program, National Taiwan University and Academia Sinica, Taipei 106, Taiwan
[6]Department of Agricultural Chemistry, National Taiwan University, Taipei 106, Taiwan

*Correspondence: andyhsu@ntu.edu.tw (C.H.H.), bmchinp@ibms.sinica.edu.tw (C.C.)

Supplementary Table S1. Dynamic parameters obtained by fitting the ^{15}N relaxation dispersion profiles of individual residues of Myb1 alone and in complex with *Tv*CyP1 to a two-site exchange process using CR72 model.

	Residue	k_{ex} (s^{-1})	*p*A (%)	$\Delta\omega$ (ppm)
Myb1 alone	THR39	579.9 ± 129.9	98.6 ± 0.9	1.26 ± 0.19
	GLU41	786.0 ± 66.2	98.1 ± 0.1	1.90 ± 0.09
	GLU42	933.6 ± 91.4	98.3 ± 0.2	1.90 ± 0.14
	LEU46	1196.4 ± 191.6	98.7 ± 1.0	1.93 ± 0.33
	GLN47	773.3 ± 289.8	99.2 ± 3.1	2.18 ± 0.44
	GLN48	2428.5 ± 193.8	95.2 ± 4.0	2.18 ± 0.40
	LEU49	1397.8 ± 404.3	98.2 ± 2.1	3.15 ± 0.58
	VAL50	3171.3 ± 1239.5	98.5 ± 3.5	3.10 ± 1.25
	ARG52	2290.1 ± 1671.0	99.7 ± 3.2	4.27 ± 1.78
	ALA55	4334.0 ± 1223.8	98.7 ± 3.9	2.53 ± 1.14
	LEU64	1894.7 ± 1981.8	99.8 ± 5.5	6.82 ± 2.60
	GLN72	1883.7 ± 2572.0	99.9 ± 5.3	5.11 ± 3.01
	CYS73	2548.1 ± 1698.3	99.8 ± 0.0	6.94 ± 2.26
	GLU75	2505.1 ± 277.7	98.2 ± 1.2	2.79 ± 0.46
	ASN78	3854.3 ± 359.7	80.0 ± 7.9	2.48 ± 0.51
	TYR80	6016.8 ± 3565.6	99.2 ± 3.3	5.97 ± 3.34
	ILE81	266.8 ± 414.9	98.7 ± 14.0	4.26 ± 0.51
	ALA84	2229.1 ± 476.7	99.1 ± 1.8	2.79 ± 0.70
	ARG86	1310.3 ± 138.4	98.2 ± 0.3	2.30 ± 0.20
	THR87	899.1 ± 274.9	99.5 ± 1.3	3.26 ± 0.33
	TRP90	1144.7 ± 112.3	99.0 ± 0.0	4.08 ± 0.13
	GLU94	243.9 ± 149.2	98.0 ± 9.8	2.42 ± 0.17
	GLN100	4117.6 ± 503.1	94.6 ± 4.7	3.08 ± 0.66
	LYS101	706.5 ± 297.3	98.8 ± 6.0	3.62 ± 0.34
	TYR102	1538.8 ± 282.6	99.0 ± 0.1	5.19 ± 0.31
	ALA103	1862.0 ± 371.9	98.7 ± 0.6	3.32 ± 0.49
	GLU104	21.9 ± 121.9	75.6 ± 15.8	3.32 ± 0.29
	TRP109	373.1 ± 157.8	98.7 ± 6.6	2.21 ± 0.16
	LYS111	2065.5 ± 345.5	98.6 ± 2.9	1.60 ± 0.62
	ILE112	785.3 ± 154.4	99.2 ± 0.1	2.12 ± 0.20
	PHE115	159.8 ± 214.6	95.8 ± 12.9	4.60 ± 0.30
	LYS117	588.6 ± 97.8	98.8 ± 0.1	2.77 ± 0.12
	ASN118	546.4 ± 108.9	98.7 ± 0.3	3.14 ± 0.12
	ARG119	128.9 ± 202.7	94.7 ± 10.1	5.10 ± 0.22
	SER120	868.2 ± 102.1	98.8 ± 0.1	3.26 ± 0.12
	ASN122	82.6 ± 139.8	92.0 ± 10.6	4.04 ± 0.23
	ASN123	589.4 ± 140.6	98.9 ± 1.5	2.84 ± 0.15
	ARG125	326.1 ± 162.4	98.3 ± 7.3	2.69 ± 0.17
	ASN126	129.7 ± 132.1	95.1 ± 11.8	3.22 ± 0.23

	ARG127	488.2 ± 182.1	98.9 ± 2.8	1.64 ± 0.24
	ILE131	3277.5 ± 415.2	95.7 ± 5.2	1.90 ± 0.69
	ARG133	3607.3 ± 387.8	97.5 ± 3.9	2.77 ± 0.64
	ARG135	574.9 ± 198.0	99.1 ± 3.4	2.55 ± 0.23
Myb1/ *Tv*CyP1	THR39	1049.4 ± 438.4	99.3 ± 2.9	2.02 ± 0.61
	GLU40	1084.7 ± 1454.0	99.8 ± 7.4	2.68 ± 1.17
	GLU41	550.3 ± 138.7	98.5 ± 1.1	2.38 ± 0.16
	GLU42	765.1 ± 192.2	98.8 ± 1.0	1.92 ± 0.27
	LEU46	1961.9 ± 401.9	96.3 ± 4.5	1.18 ± 0.60
	GLN47	417.7 ± 211.6	98.3 ± 6.8	1.29 ± 0.34
	LEU49	1540.0 ± 634.6	98.3 ± 4.3	3.11 ± 0.87
	VAL50	5697.1 ± 3264.4	99.0 ± 2.1	6.21 ± 2.92
	ARG52	1200.3 ± 1208.2	99.7 ± 7.4	4.73 ± 1.43
	ALA55	2605.1 ± 1313.0	99.6 ± 0.6	4.57 ± 1.31
	TRP58	3414.0 ± 4616.3	99.6 ± 3.7	5.00 ± 3.46
	ARG71	2200.4 ± 2888.3	99.9 ± 5.1	5.55 ± 3.03
	GLU75	2047.6 ± 326.9	98.4 ± 1.4	2.98 ± 0.46
	TYR80	3223.0 ± 3239.6	99.3 ± 5.3	4.81 ± 3.26
	ALA84	1486.5 ± 664.6	99.2 ± 1.5	4.11 ± 0.70
	LEU85	554.0 ± 120.3	98.0 ± 1.3	2.12 ± 0.15
	ARG86	922.2 ± 200.1	98.1 ± 0.5	2.25 ± 0.29
	ASP88	535.4 ± 194.6	98.7 ± 2.3	1.58 ± 0.28
	TRP90	3412.3 ± 2627.9	99.5 ± 1.7	5.58 ± 2.42
	SER91	1365.4 ± 986.5	99.5 ± 4.9	4.38 ± 1.12
	GLU94	344.3 ± 301.8	98.9 ± 11.7	2.45 ± 0.39
	ASP95	2450.5 ± 6401.2	99.9 ± 12.9	9.01 ± 3.91
	MET96	1723.3 ± 2157.2	99.7 ± 8.4	2.78 ± 2.31
	LEU97	120.3 ± 230.4	97.2 ± 13.3	2.10 ± 0.35
	TYR102	753.2 ± 422.7	98.8 ± 9.9	4.87 ± 0.42
	ALA103	1714.2 ± 650.5	99.0 ± 2.0	3.99 ± 0.66
	TRP109	80.3 ± 812.8	97.1 ± 16.4	4.87 ± 1.04
	ASN118	198.7 ± 220.8	97.4 ± 11.7	3.26 ± 0.30
	SER120	909.4 ± 260.8	99.0 ± 1.2	2.88 ± 0.31
	ASN123	444.7 ± 358.6	99.0 ± 10.9	2.62 ± 0.46
	ARG125	6709.4 ± 1546.4	99.7 ± 0.0	8.66 ± 1.46
	ARG127	428.4 ± 240.4	98.8 ± 5.9	1.52 ± 0.34
	TRP128	3971.1 ± 1776.6	98.0 ± 3.5	3.42 ± 1.62
	ILE131	2226.8 ± 561.4	98.8 ± 1.1	3.58 ± 0.70
	ARG133	4484.2 ± 2199.5	98.3 ± 4.2	3.58 ± 1.71
	ALA136	659.3 ± 257.1	99.2 ± 4.5	2.20 ± 0.33
	LYS137	388.5 ± 272.0	99.1 ± 10.5	2.23 ± 0.36
	GLN139	629.7 ± 253.6	97.8 ± 7.4	0.72 ± 0.38

Supplementary Table S2: Dynamic parameters obtained by fitting the ^{15}N relaxation dispersion profiles of individual residues of Myb1 alone and in complex with *Tv*CyP1 to a two-site exchange process using TSMFK01 model.

	Residue	k_{ex} (s^{-1})	$\Delta\omega$ (ppm)
Myb1 alone	LYS56	1.23 ± 0.10	5.22 ± 0.41
	ILE61	2.34 ± 0.14	4.95 ± 0.29
	ARG68	2.85 ± 0.21	6.89 ± 0.45
	ARG74	0.67 ± 0.19	10.68 ± 1.92
	ASN79	5.51 ± 0.67	8.17 ± 0.95
	GLU93	3.73 ± 0.11	3.54 ± 0.11
	ASP95	1.46 ± 0.26	6.35 ± 0.93
	MET96	0.91 ± 0.14	7.04 ± 1.03
	LEU98	1.63 ± 0.45	8.47 ± 0.72
	ASP99	3.20 ± 0.36	2.30 ± 0.30
	TYR105	5.57 ± 0.23	6.67 ± 0.19
	LYS108	7.37 ± 0.20	8.74 ± 0.17
	ASN110	5.84 ± 0.17	7.38 ± 0.19
	SER113	5.67 ± 0.16	5.47 ± 0.16
	LEU116	1.74 ± 0.14	8.27 ± 0.56
	ALA132	2.88 ± 0.25	2.24 ± 0.20
	HIS134	1.82 ± 0.14	2.90 ± 0.24
Myb1/ *Tv*CyP1	TYR53	1.82 ± 0.26	5.26 ± 0.81
	LYS56	1.91 ± 0.17	6.47 ± 0.56
	ILE61	2.34 ± 0.21	4.79 ± 0.47
	SER62	1.96 ± 0.25	7.30 ± 0.84
	LEU64	1.60 ± 0.20	4.50 ± 0.65
	GLN72	1.16 ± 0.21	4.93 ± 1.02
	CYS73	2.35 ± 0.31	5.77 ± 0.78
	ARG74	1.52 ± 0.31	4.34 ± 0.98
	TRP77	1.50 ± 4.45	6.42 ± 2.91
	ASN78	24.77 ± 1.73	6.73 ± 0.39
	ASN79	5.57 ± 1.30	8.07 ± 1.64
	GLU93	3.05 ± 0.23	2.80 ± 0.23
	LEU98	2.58 ± 0.56	8.75 ± 1.20
	ASP99	3.71 ± 0.51	8.71 ± 1.07
	LYS101	4.04 ± 0.35	4.77 ± 0.45
	GLU104	3.71 ± 0.23	5.16 ± 0.32
	TYR105	2.99 ± 0.26	7.16 ± 0.59
	LYS108	5.13 ± 0.27	9.10 ± 0.33
	ASN110	2.91 ± 0.33	7.60 ± 0.74
	LYS111	1.25 ± 0.22	7.38 ± 1.19
	ILE112	3.16 ± 0.22	2.93 ± 0.23
	SER113	3.24 ± 0.30	7.04 ± 0.63

	PHE115	5.05 ± 0.27	5.31 ± 0.29
	LYS117	4.60 ± 0.22	3.11 ± 0.16
	ARG119	5.30 ± 0.20	6.00 ± 0.22
	ASP121	2.32 ± 6.87	1.60 ± 0.29
	ASN122	3.86 ± 0.26	4.80 ± 0.30
	ASN126	4.25 ± 0.28	4.81 ± 0.27
	ALA132	1.64 ± 0.38	8.56 ± 1.71
	HIS134	1.57 ± 0.37	12.68 ± 1.66
	ARG135	2.60 ± 0.29	5.18 ± 0.60
	SER141	0.46 ± 28.75	2.10 ± 1.50

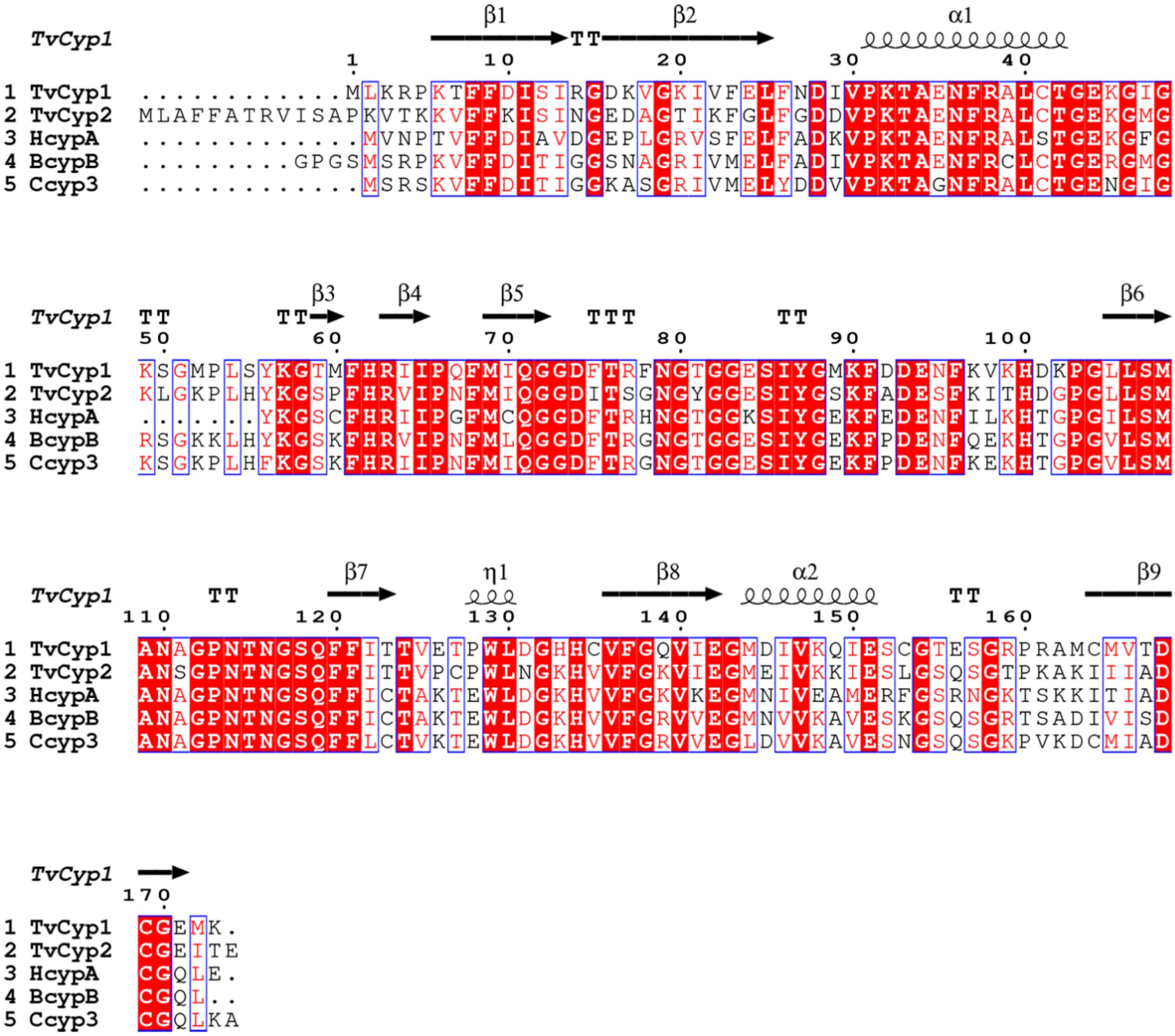

Supplementary Fig. S1. **Multiple Sequence alignment of *Tv*CyP1.** Sequence alignment of *Tv*CyP1 with cyclophilin 2 (*Tv*CyP2) from *T. vaginalis*, Human cyclophilin A (*H*cypA; PDB: 1OCA), cyclophilin B (*B*cypB) from *B. malayi* (PDB: 4JCP) and cyclophilin 3 (*C*cyp3) from *C. elegans* (PDB: 1DYW), showing 71%, 62%, 68% and 70% sequence identity, respectively. The figure was produced by using Clustal Omega and ESPRIPT. Secondary structure of *Tv*CyP1 is shown on top.

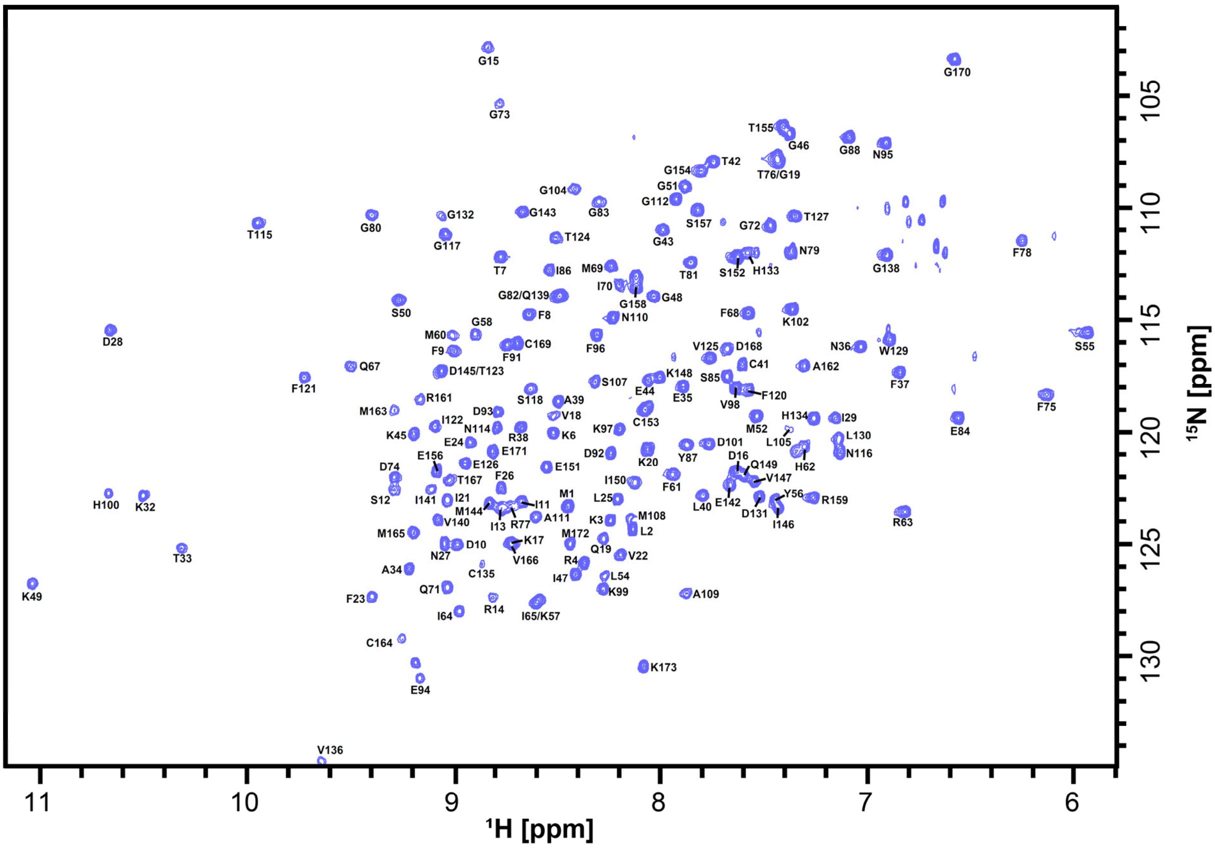

Supplementary Fig. S2. **Backbone Assignment of *Tv*CyP1.** 2D ^{1}H-^{15}N TROSY-HSQC spectrum of ^{15}N-labeled *Tv*CyP1 showing assignments as indicated by residue name and number.

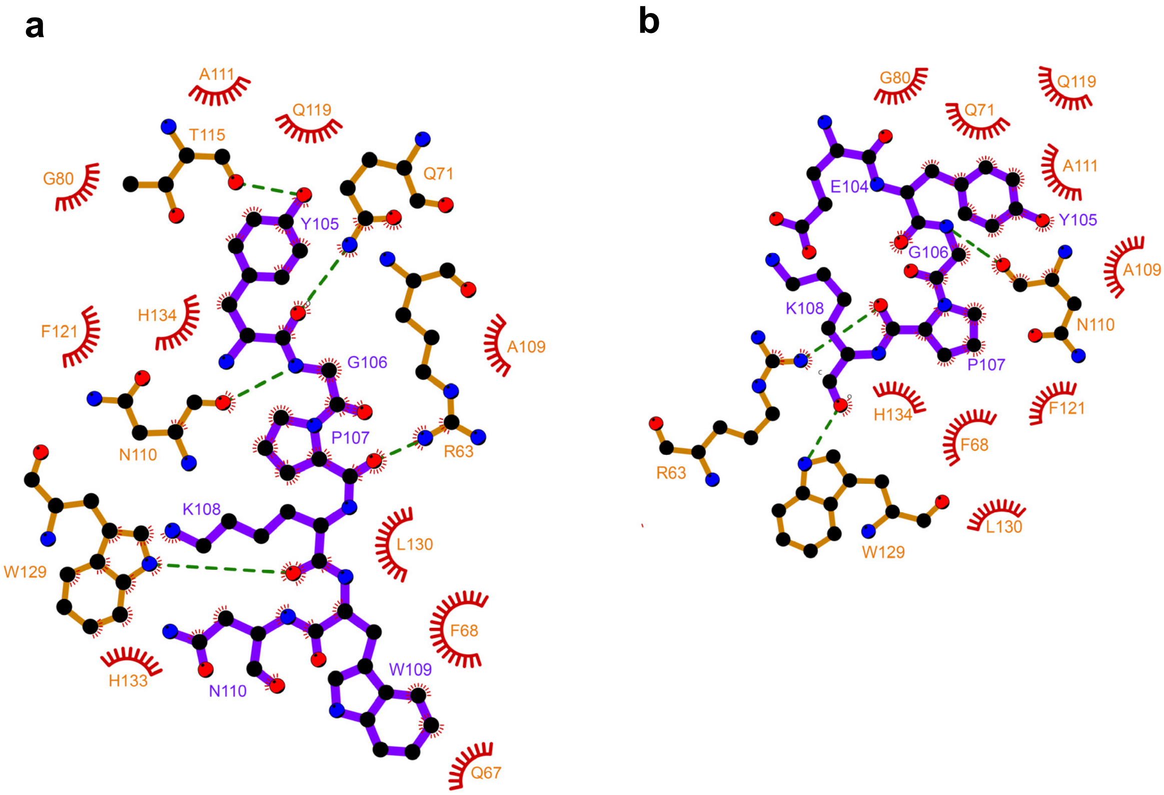

Supplementary Fig. S3. 2D diagram showing Myb1 peptide interactions with *Tv*CyP1 by LigPlot+. (**a**) The interactions of protomer I of *Tv*CyP1 with ^{105}YGPKWN^{110} and (**b**) the interactions of protomer II of *Tv*CyP1 with ^{104}EYGPK^{108}. Hydrogen bonds are shown as green dashed lines and hydrophobic interactions of the Myb1 peptide with *Tv*CyP1 are shown as red spokes.

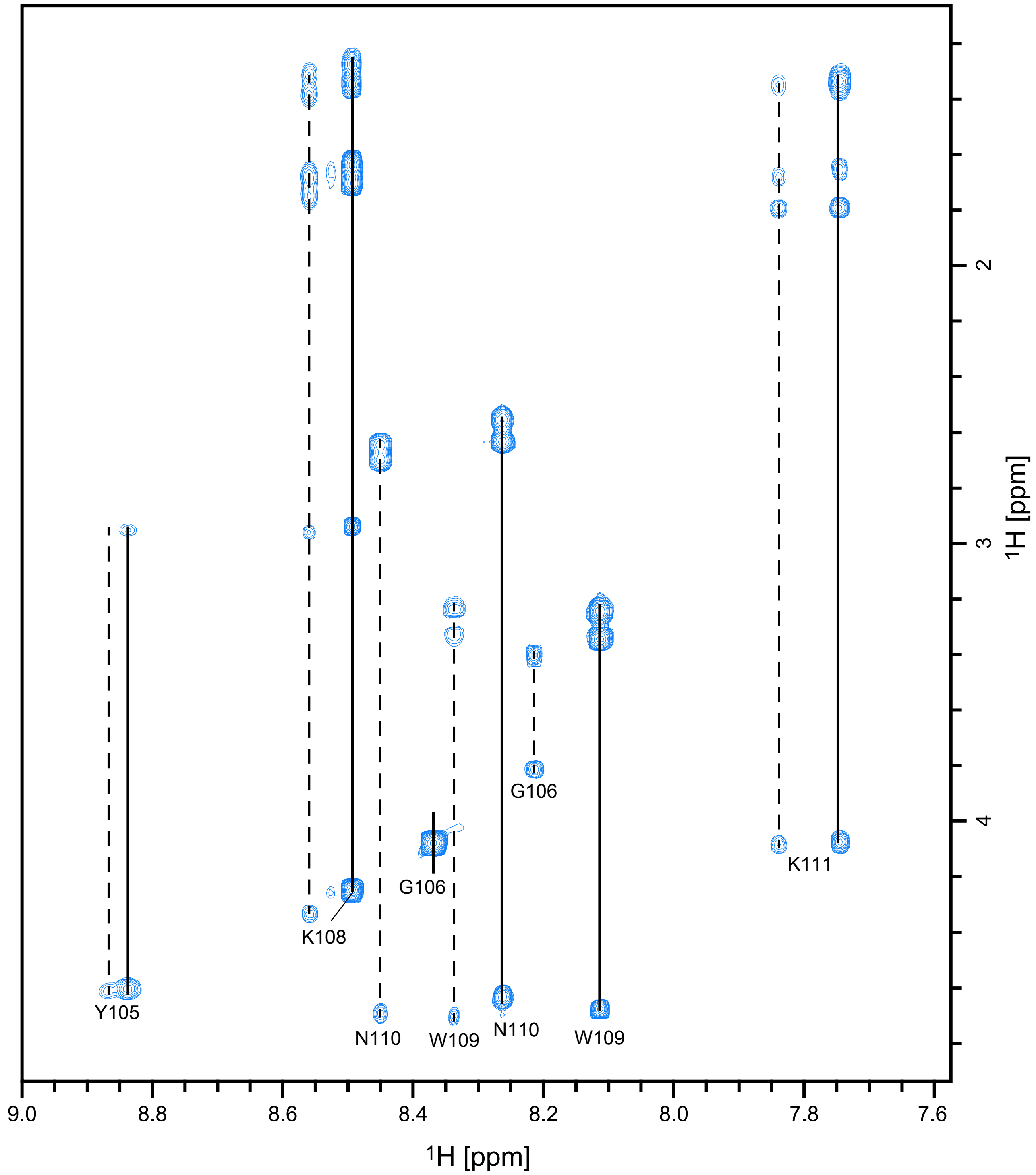

Supplementary Fig. S4. Finger print region of 2D total correlated spectroscopy (TOCSY) spectrum of Myb1$^{104\text{-}110}$ peptide at 280K. The peptide (4.4 mM) was prepared in buffer containing 20 mM NaH_2PO_4 and 50 mM NaCl, at pH 6.0. Separate resonance signals for *cis* and *trans* conformations were observed for residues from Y105 to K111 and were connected with dashed and solid lines, respectively.

a

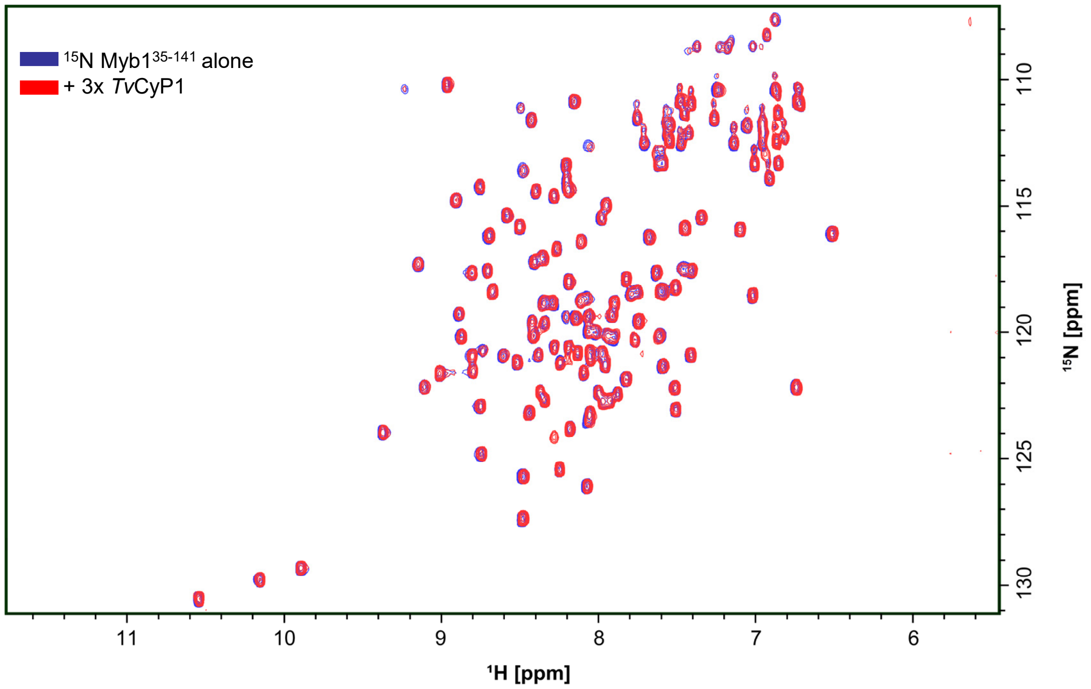

b

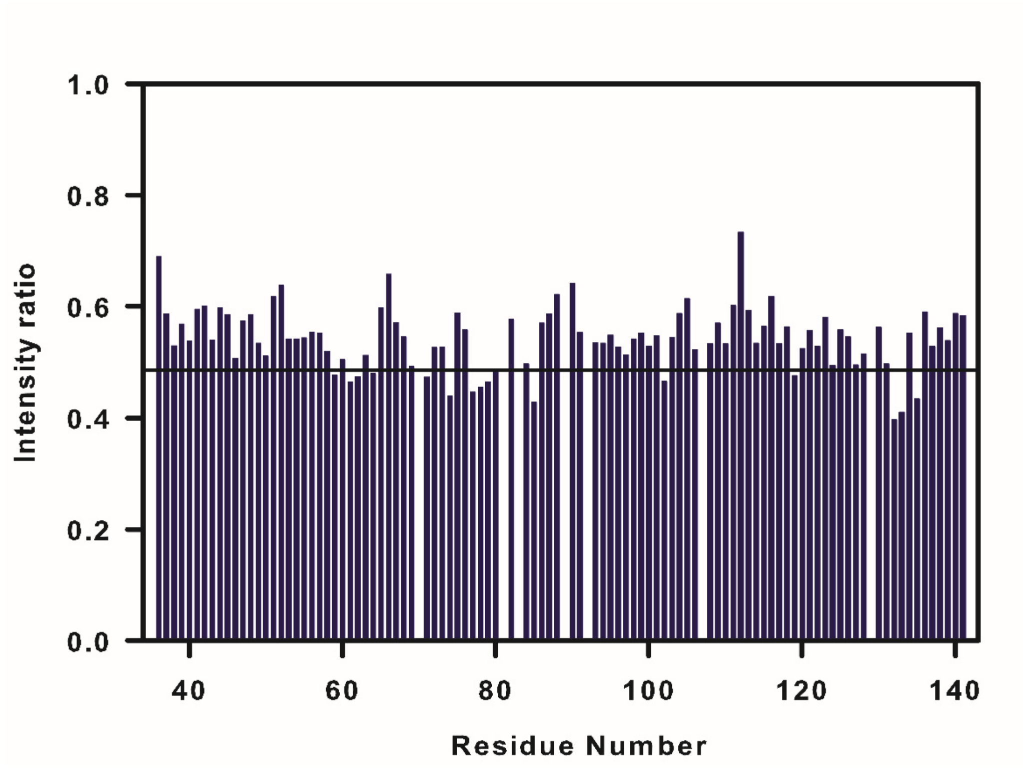

Supplementary Fig. S5. **NMR titration of unlabelled *Tv*CyP1 on ^{15}N labelled Myb1$^{35\text{-}141}$.** (**a**) 2D ^{1}H-^{15}N HSQC spectrum of ^{15}N-labeled Myb1$^{35\text{-}141}$ (blue) overlapped with the spectrum acquired during the titration of unlabelled *Tv*CyP1 in the ratio 1:3 (red). (**b**) Plot of the ratio of intensities of Myb1$^{35\text{-}141}$ peaks in the presence and absence of *Tv*CyP1 against the residue number. Horizontal black line signifies average minus one SD.

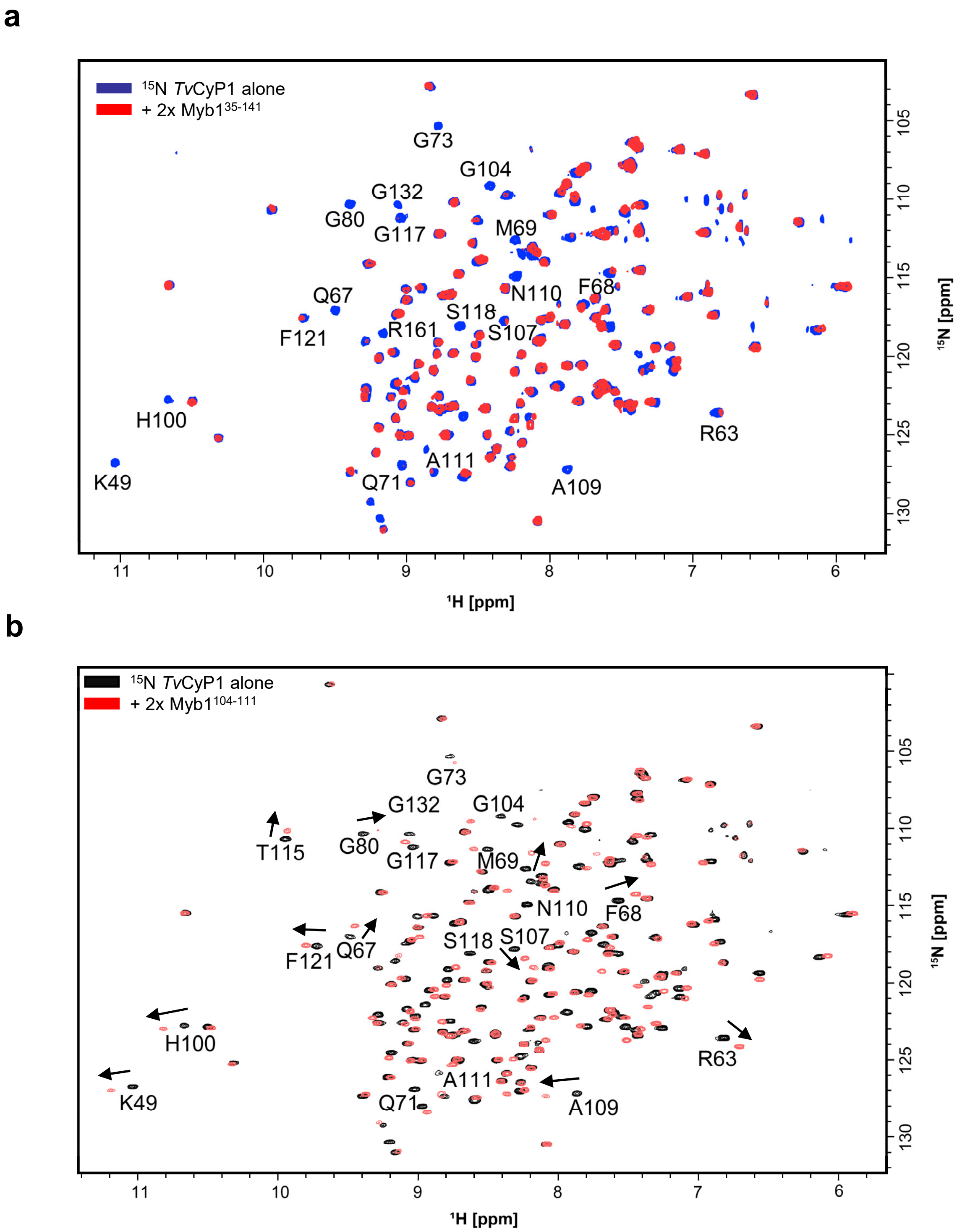

Supplementary Fig. S6. NMR titrations on *Tv*CyP1 with Myb1$^{35\text{-}141}$ and Myb1$^{104\text{-}111}$. (**a**) 2D ^{1}H-^{15}N TROSY-HSQC spectrum of 100 µM ^{15}N-labeled *Tv*CyP1 (blue) overlapped with the spectrum acquired during the titration of unlabeled Myb1$^{35\text{-}141}$ in the ratio of 1:2 (red). Residues showing significant line-width broadening are labeled. (**b**) 2D ^{1}H-^{15}N TROSY-HSQC spectrum of ^{15}N-labeled *Tv*CyP1 (black) overlapped with the spectrum acquired during the titration of unlabeled Myb1 peptide, Myb1$^{104\text{-}111}$ (red) in the ratio of 1:2. Residues showing significant CSPs are labelled.

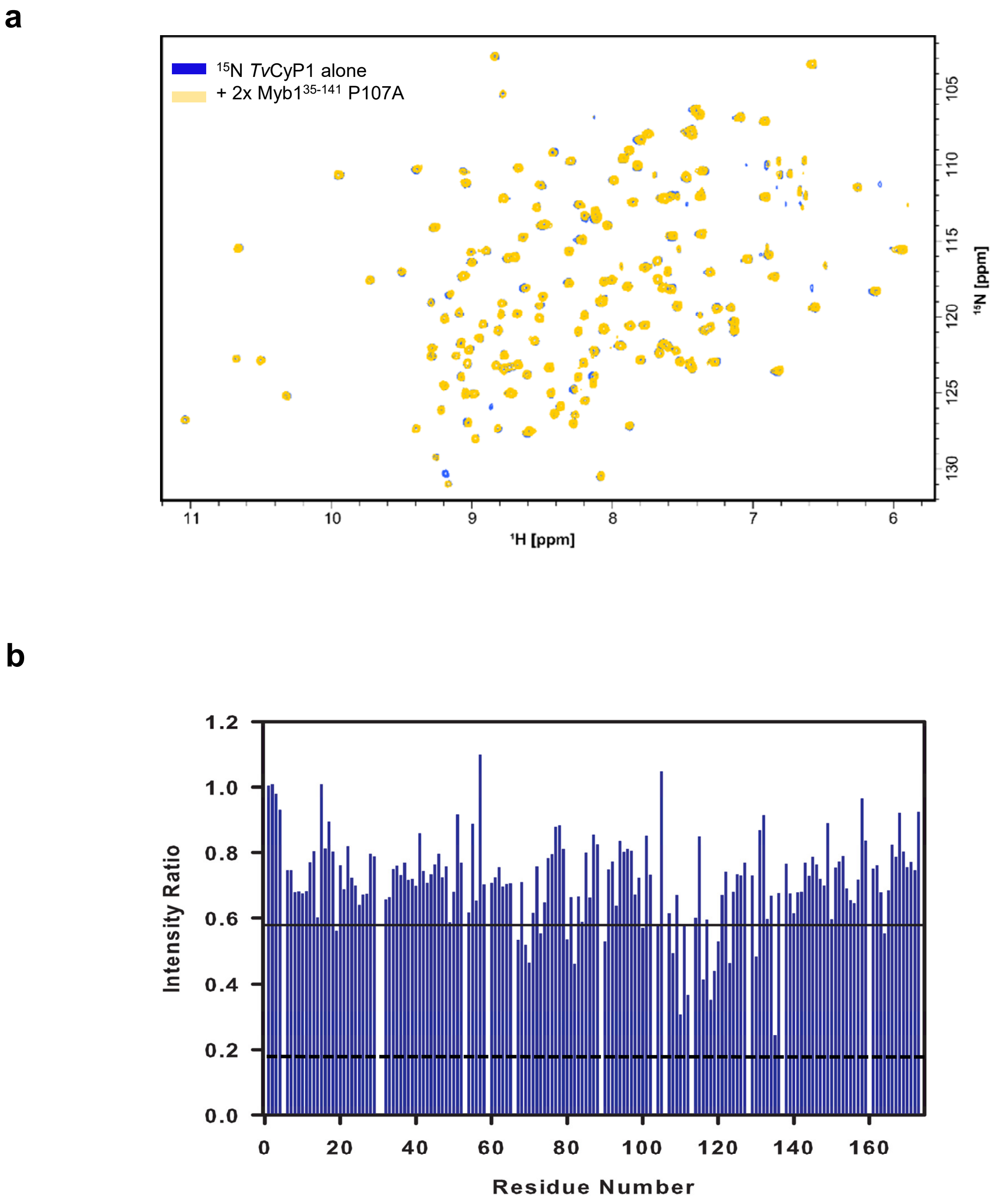

Supplementary Fig. S7. NMR titration on ^{15}N *Tv*CyP1 with unlabeled Myb1$^{35\text{-}141}$ mutant P107A. (**a**) 2D ^{1}H-^{15}N TROSY- HSQC spectrum of 100 µM ^{15}N-labeled *Tv*CyP1 (blue) overlapped with spectrum acquired during titration of unlabeled P107A-Myb1$^{35\text{-}141}$ mutant in the ratio 1:2 (yellow). (**b**) Plot of the ratio of intensities of *Tv*CyP1 peaks in the presence and absence of P107A-Myb1$^{35\text{-}141}$ against the residue number. Black horizontal line signifies average minus one SD. Dashed line signifies average minus one SD for *Tv*CyP1- Myb1$^{35\text{-}141}$ titration.
